# Predictive value of ultrasound findings for high-grade vesicoureteral reflux in children: a retrospective cross-sectional study in Saudi Arabia

**DOI:** 10.3389/fped.2026.1932063

**Published:** 2026-08-19

**Authors:** Abdulaziz S. Aljibali

**Affiliations:** Department of Radiology, College of Medicine, Qassim University, Qassim, Saudi Arabia

**Keywords:** computer assisted diagnosis, diagnostic imaging, pediatric, ultrasound, vesicoureteral reflux, voiding cystourethrography

## Abstract

**Background:**

Voiding cystourethrography (VCUG) remains the gold standard for diagnosing vesicoureteral reflux (VUR) but is invasive, involves ionizing radiation, and may cause considerable discomfort in children. Ultrasound offers a non-invasive alternative, although its ability to predict severe reflux remains uncertain. This study evaluated the predictive value of ultrasound findings for the detection of high-grade VUR and identified sonographic predictors associated with severe reflux in children.

**Methods:**

A retrospective cross-sectional study was conducted involving pediatric patients aged 0–14 years who underwent both renal and bladder ultrasound and VCUG between January 2018 and December 2024 in tertiary and secondary healthcare facilities in the Central Region of Saudi Arabia. Ultrasound variables including hydronephrosis severity, ureteral dilatation, cortical thinning, renal scarring, and bladder abnormalities were compared with VCUG findings. Chi-square analysis, multivariate logistic regression, receiver operating characteristic (ROC) analysis, and diagnostic performance measures were performed.

**Results:**

A total of 406 children were included, with high-grade VUR identified in 50 patients (12.3%) on VCUG. Severe right and left hydronephrosis, ureteral dilatation, right cortical thinning, right renal scarring, and bladder distention were significantly associated with high-grade VUR (*p* < 0.05). Multivariate analysis identified left hydronephrosis (OR = 4.54), right hydronephrosis (OR = 4.18), bladder distention (OR = 4.18), and right renal scarring (OR = 3.76) as independent predictors. The combined ultrasound model demonstrated good discriminative ability (AUC = 0.824), sensitivity of 78.0%, specificity of 76.4%, positive predictive value of 31.7%, negative predictive value of 96.1%, and overall accuracy of 76.6%.

**Conclusion:**

Multiparametric ultrasound is an effective screening tool for excluding high-grade VUR and may reduce unnecessary VCUG examinations but should not be seen as a replacement for VCUG.

## 1 Background

Vesicoureteral reflux (VUR) is one of the most common congenital urinary tract abnormalities in children and is characterized by the retrograde flow of urine from the bladder into the ureters and kidneys due to an incompetent vesicoureteral junction. Although mild cases may resolve spontaneously, high-grade VUR is clinically significant because persistent reflux predisposes children to recurrent urinary tract infections (UTIs), renal scarring, hypertension, and progressive chronic kidney disease. Early identification of high-grade reflux is therefore essential to prevent irreversible renal damage and preserve long-term kidney function. Zhu et al. (1) reported that severe VUR is strongly associated with deterioration in renal function and chronic renal injury, emphasizing the importance of prompt diagnosis and intervention.

Voiding cystourethrography (VCUG) remains the gold standard for diagnosing and grading VUR because it directly demonstrates reflux during bladder filling and voiding. However, its routine use is limited by several disadvantages, including radiation exposure, urethral catheterization, procedural discomfort, and increased healthcare costs. These limitations have stimulated interest in identifying non-invasive imaging techniques that can accurately predict high-grade reflux while minimizing patient discomfort and radiation exposure.

Ultrasound has become the preferred initial imaging modality for evaluating pediatric urinary tract abnormalities because it is safe, inexpensive, readily available, and free of ionizing radiation. Conventional renal and bladder ultrasound can detect structural abnormalities associated with severe reflux, including hydronephrosis, ureteral dilatation, cortical thinning, renal scarring, bladder wall abnormalities, and renal asymmetry. Öztürk et al. (2) demonstrated that these sonographic features are valuable indicators of clinically significant reflux. Similarly, Gemmell et al. (3) found that ureteric jet angle measurement improves the diagnostic performance of ultrasound, while Enayati Zadeh et al. (4) reported that colour Doppler assessment of ureteric jet asymmetry enhances reflux detection.

Recent technological developments have expanded the diagnostic potential of ultrasound. Ultrasound elastography allows assessment of renal parenchymal stiffness and may facilitate early detection of reflux nephropathy before irreversible structural damage develops. Kocaoglan et al. (5) demonstrated that elastography provides valuable information regarding renal involvement in children with VUR. Likewise, contrast-enhanced voiding urosonography (ceVUS) has emerged as a highly sensitive, radiation-free alternative to VCUG. Hu et al. (6) reported that ceVUS provides excellent diagnostic accuracy for detecting and grading VUR while avoiding ionizing radiation. Furthermore, artificial intelligence (AI) and machine learning approaches are increasingly being integrated into diagnostic imaging as Alqaraleh et al. (7) developed predictive models capable of classifying VUR severity using ultrasound-derived features, suggesting that AI may improve risk stratification and reduce reliance on invasive investigations.

Delayed diagnosis of high-grade VUR may result in permanent renal damage extending into adulthood. De Oliveira Tavares et al. (8) highlighted the long-term consequences of reflux nephropathy and emphasized the importance of early diagnosis and monitoring. Similarly, Laleoğlu et al. (9) demonstrated that combining ultrasound findings with clinical variables improves the prediction of severe reflux, supporting a more individualized diagnostic approach.

Although Saudi Arabia has made significant progress in pediatric healthcare and diagnostic imaging, congenital urinary tract anomalies and pediatric UTIs remain important health concerns. In line with Saudi Vision 2030, there is increasing emphasis on evidence-based practice, digital health, and minimizing unnecessary invasive procedures. However, the predictive value of ultrasound for detecting high-grade VUR among Saudi children remains insufficiently studied. Therefore, this study aimed to evaluate the diagnostic performance of renal and bladder ultrasound in predicting high-grade VUR using VCUG as the reference standard. The findings are expected to support safer, evidence-based diagnostic pathways and improve clinical management of children with suspected VUR.

## 2 Methodology

### 2.1 Research design

This study adopted a retrospective cross-sectional design. The study was performed in accordance with the World Medical Association Declaration of Helsinki: Ethical principles for medical research involving human participants and followed the International Committee of Medical Journal Editors (ICMJE) recommendations for the conduct, reporting, editing and publication of scholarly work in medical journals.

### 2.2 Study period

This retrospective study included pediatric patients who underwent renal and bladder ultrasound and VCUG between January 2018 and December 2024. The retrospective chart review, data extraction, verification, statistical analysis, and manuscript preparation were conducted between May 2026 and July 2026 after ethics approval. Thus, the former period represents the timeframe of the patient records included in the study, whereas the latter represents the period during which the research activities were performed. A long study period is required because the previous occurrence of high-grade vesicoureteral reflux in the general paediatric population in the study area is relatively low. The extended study period ensured that seasonal changes, referral dynamics, and institutional alterations of imaging procedures have been included. This period has also enabled the employment of large enough and representative data, which enhances the external validity of the results. The chosen period is consistent with other retrospective imaging projects on the unit of paediatric urology that tend to use several-year datasets to increase reliability and statistical power.

### 2.3 Research data collection method

Data were collected through a structured review of existing clinical records. Electronic medical records, radiology information systems, and picture archiving and communication systems (PACS) were searched to retrieve information. To minimize variability regarding data recording, a standard data extraction form was created. Relevant clinical and imaging data (ultrasound findings: hydronephrosis, ureteral dilatation, renal cortical abnormalities), VCUG results, demographic variables, and clinical history were extracted. A second reviewer independently reviewed a subset of records to increase accuracy, and any differences were resolved by consensus.

### 2.4 Sampling frame and sample size

The study sample comprised children under 14 years of age who have both been subjected to renal ultrasound and VCUG to examine the issue of abnormalities in the Urinary tract. All eligible children who were evaluated in the chosen healthcare facilities throughout the study period were included the study.

A consecutive sampling method was used and sample size has been determined using the standard formula for cross-sectional studies:

$$n=\frac{Z^{2}\;p(1-p)}{d^{2}}$$

where: Z = 1.96, corresponding to a 95% confidence level; *p* = 0.50, estimated prevalence of high-grade vesicoureteral reflux; d = 0.05, margin of error (precision level)

$$n=\frac{\left(1.96{)}^{2}\times 0.5(1-0.5\right)}{{\left(0.05\right)}^{2}}$$

$$n=\frac{3.8416\times 0.25}{0.0025}$$

$$n=\frac{0.9604}{0.0025}$$

n=384.16

The required sample size was calculated to be 384 participants. The final sample size of about 400 pediatric patients is enlarged to guarantee adequate statistical power and account for incompleteness, missing data, or exclusion of medical records. A prevalence estimate of 0.50 was used because no reliable estimate of high-grade VUR predicted by ultrasound in the Saudi pediatric population was available. A total of 406 patients were ultimately included.

### 2.5 Inclusion and exclusion criteria

The target population comprises all the pediatric patients aged 0–14 years, diagnosed or suspected of having urinary tract infection or vesicoureteral reflux in tertiary and secondary care in the Central Region of Saudi Arabia. This population was chosen since VUR occurs most frequently in infants and young children, especially those who present with febrile UTIs.

Pediatric patients were excluded if they were not diagnosed or suspected of having urinary tract infection or vesicoureteral reflux in tertiary and secondary care in the Central Region of Saudi Arabia. Patients older than 14 years were also excluded.

### 2.6 Reliability and validity

To enhance reliability, uniform data extraction methods were adopted, and selected records were subjected to data verification by dual review. This minimized inter-observer differences in data collection. Ultrasound reporting consistency was ensured by relying on radiology reports prepared by trained pediatric radiologists with standardized imaging protocols.

VCUG was used as the gold standard in diagnosis and grading of VUR to ensure validity. This improves criterion validity of the study. Moreover, the construct validity was achieved by incorporating ultrasound variables that have earlier been validated in the literature, like Taş et al. (10).

### 2.7 Data analysis

Data were analyzed using IBM SPSS Statistics. Demographic and clinical characteristics of the study population were summarised using descriptive statistics. Continuous variables were summarised by means and standard deviations, and categorical variables summarised in terms of frequencies and percentages.

Associations between ultrasound findings and high-grade VUR were tested using inferential statistical tests. Categorical variables were analysed by chi-square tests, whereas independent *t*-tests or non-parametric variants were selected according to the data distribution and analysed for continuous variables.

Multivariate logistic regression analysis was performed to determine independent predictors of high-grade VUR. Measures of strength of association were reported using odds ratios with 95% confidence intervals. The analysis of receiver operating characteristic (ROC) curves was performed to evaluate diagnostic performance of ultrasound findings, and the area under the curve (AUC) was utilized to quantify its predictive accuracy.

VCUG was used as the reference standard to calculate the accuracy, sensitivity, specificity, positive predictive value, and negative predictive value.

### 2.8 Ethical consideration

The Research Ethics Committee at Prince Sultan Military Medical City provided ethical approval before data were collected. As this has been a retrospective study, a waiver of informed consent was requested and granted. All patient records were anonymized and coded to ensure confidentiality. In addition, no identifiable patient information was collected or disclosed. The storage of data was secured by the use of password-enabled systems that are only accessible by the research team. The study adhered to both institutional and international research ethics on research involving human subjects. This ethical framework was validated since there is low risk in the retrospective studies, as no direct interaction or intervention on a patient was done.

## 3 Results

### 3.1 Demographic characteristics of the study population

A total of 406 patients were included in the final analysis after meeting the inclusion criteria. The major demographic and clinical features of the study sample are summed up in Table 1.

**Table 1:** Demographic and clinical characteristics of the study population (*N* = 406).

Variable	n (%)	Frequency
Gender		
Male	65.0%	264
Female	35.0%	142
Age Group		
Infants (<12 months)	63.3%	257
1–5 Years	22.9%	93
6–12 Years	13.8%	56
Clinical Indication		
Antenatal hydronephrosis	45.3%	184
UTI	21.4%	87
Other Indications	33.3%	135
UTI History		
Yes	16.3%	66
No	83.7%	340
Age—Mean ± SD	28.9 ± 41.4 months	

SD, Standard Deviation. Percentages are calculated from the total sample (*N* = 406). Age is presented in months.

Most participant were males, with 264 male patients (65.0%) and 142 female patients (35.0%), consistent with previously reported pediatric urology referral patterns. The mean age was 28.9 months with a standard deviation of 41.4 months, and it implies that most of the patients were infants and toddlers. Participant ages ranged from 1 to 153 months. Specifically, the proportion of patients below twelve months of age (257, 63.3%) is in line with the fact that a high percentage of referrals to follow-ups of antenatal hydronephrosis occurred during early infancy. Antenatal hydronephrosis was the most common referral factor (184 cases, 45.3%), then originating from other indications of UTIs like recurrent UTI, urinary obstruction, and non-specific urinary symptoms (135 cases, 33.3%), and primary diagnosis of a urinary tract infection (87 cases, 21.4%). Sixty-six patients (16.3%) had a previous history of UTI, and 340 patients (83.7%) had no UTI history recorded **(**Table 1). These demographic results are valuable in the interpretation of ultrasound and VCUG outcomes in a population of mostly young infants who are sent because of what is believed to be urinary tract abnormalities.

### 3.2 Descriptive analysis of ultrasound findings

#### 3.2.1 Hydronephrosis severity (right and left sides)

The Society for Fetal Urology (SFU) grading system was utilized to grade hydronephrosis on the right and left sides on a scale of 0–4. The majority of patients exhibited no hydronephrosis on the right (Grade 0 = 35.5%) or slight hydronephrosis on the right (Grade 1 = 34.7%) (Table 2)**.** Only 24 patients had severe hydronephrosis (Grade 4). The average grade of hydronephrosis of the right duct was 1.15 (SD ± 1.17) (Table 2). On the left-hand side, the distribution of the grades was more balanced, and Grade 2 was the most common finding (26.4%). The 32 patients had left-sided Grade 4 hydronephrosis (7.9%), and 1.55 (SD ± 1.25) was significantly higher than the right hydronephrosis grade. This side-to-side difference may be clinically relevant and could help partly clarify subsequent links to high-grade VUR. The total rates of moderate to severe hydronephrosis (Grades 3–4) were 14.3% on the right side and 22.7% on the left side, which suggests that most individuals in this cohort had a significant presence of progressive renal pelvis dilation (Table 2). Table 2 shows the frequency distribution of hydronephrosis grades in the two kidneys.

**Table 2:** Distribution of various ultrasound findings (*N* = 406).

Hydronephrosis grade	Right side (n)	Right (%)	Left side (*n*)	Left (%)
Grade 0 (None)	144	35.5%	104	25.6%
Grade 1 (Mild)	141	34.7%	102	25.1%
Grade 2 (Moderate)	63	15.5%	107	26.4%
Grade 3 (Moderately Severe)	34	8.4%	60	14.8%
Grade 4 (Severe)	24	5.9%	32	7.9%
Mean ± SD	1.15 ± 1.17		1.55 ± 1.25	

Ureteral Dilatation	Right (n)	Right (%)	Left (n)	Left (%)
Present (Yes)	40	9.9%	45	11.1%
Absent (No)	366	90.1%	361	88.9%
Total	406	100%	406	100%

Finding	Right (n)	Right (%)	Left (n)	Left (%)
Cortical Thinning				
Present	27	6.7%	31	7.6%
Absent	379	93.3%	375	92.4%
Renal Scarring				
Present	19	4.7%	15	3.7%
Absent	387	95.3%	391	96.3%
Kidney Size				
Normal	323	79.6%	319	78.6%
Small	42	10.3%	40	9.9%
Large	41	10.1%	47	11.6%

Bladder Finding	Present (n)	Present (%)	Absent (n)	Absent (%)
Bladder Wall Thickening	154	37.9%	252	62.1%
Bladder Distention (on US)	283	69.7%	123	30.3%

Yes, Ureteral dilatation confirmed on ultrasound report. No, Absent on imaging. Note. Grades 0–4 follow the SFU classification. Mean ± SD values reflect continuous severity scores. Note. Findings are reported bilaterally where applicable. Size categories are Normal, Small, and Large based on age-adjusted nomograms. Note. Bladder findings were assessed on the ultrasound examination performed prior to VCUG.

#### 3.2.2 Ureteral dilatation

Ureteral dilatation was observed in 40 patients on the right side (9.9%) and 45 patients on the left side (11.1%) (Table 2). These proportions are moderate in general; ureteral dilatation is a clinically important finding since it can be an indication of obstruction or reflux at the ureterovesical junction. This small increase in rate on the left side corresponds to the concern with the higher mean grade of hydronephrosis on that side. Ureteral dilatation and hydronephrosis in VUR assessment may indicate high-grade reflux and resultant alterations in the collecting system.

#### 3.2.3 Cortical thinning, renal scarring, and kidney size

The right side (6.7% of 27 patients) and the left side (7.6% of 31 patients) were found to have cortical thinning, indicating early or established parenchymal damage due to recurrent reflux or obstruction (Table 2). Renal scarring is a later manifestation of renal parenchymal injury and was observed in 19 patients on the right (4.7%) and 15 patients on the left (3.7%) (Table 2). Although scarring was quite rare in this cohort, it is of particular interest due to its indication of a history of previous pyelonephritis or unremitting untreated high-grade reflux. As to kidney size, most of the patients on both sides had normally sized kidneys, with 79.6 on the right and 78.6 on the left falling within normal measurements. In both cases, 42 right and 40 left-sided kidneys were observed with small size, which can be taken as evidence of long-term injury or as the result of hypoplasia during development. There were 41 right-sided patients and 47 left-sided patients in which enlarged kidneys, which might indicate obstruction or compensatory hypertrophy, were observed.

#### 3.2.4 Bladder abnormalities

Bladder wall thickening was observed in 154 patients (37.9%) (Table 2). This observation might indicate functional adaptations due to increased bladder pressures or frequent bladder inflammation. The most commonly reported bladder finding in this study was bladder distention at the time of ultrasound in 283 patients (69.7%) (Table 2). Notably, bladder distention may partly be a physiological finding at the time of ultrasound examination, particularly in infants, but it was also evaluated later in relation to VCUG findings of bladder capacity and reflux grade.

### 3.3 Prevalence of high-grade vesicoureteral reflux using VCUG

Based on VCUG findings, which served as the reference standard for VUR diagnosis and grading in this study, 50/406 patients (12.3%; high-grade VUR (Grades IV–V) were observed. In individuals with known VUR, bilateral reflux was noted in the majority of those patients (57 patients, 14.0% of all individuals), with only 17 patients (4.2% of all individuals) having right-sided reflux and 27 patients (6.6% of all individuals) having left-sided reflux. VCUG was not associated with any grade of VUR, confirming that VCUG remains the reference standard, but pathological reflux is found in a substantial proportion of patients.

### 3.4 Association between ultrasound findings and high-grade VUR: Chi-square analysis

Chi-square tests of independence were conducted to determine the statistical relation between the individual ultrasound results and the existence of high-grade VUR (Grades IV–V).

Severe hydronephrosis (Grade ≥3) on the right side was present in 18 out of 50 high-grade VUR patients (36.0%) compared to only 40 out of 356 non-high-grade VUR patients (11.2%) (Table 3). According to the chi-square test, the association was found to be statistically significant (*χ*^2^ = 19.982, df = 1, *p* = 0.0000). Likewise, on the left side, hydronephrosis Grade 3 was present in 25 of high-grade VUR patients (50.0%), and in 68 of patients without a high-grade VUR (19.1%), and this is also a way of establishing a significant result (*χ*^2^ = 21.986, df = 1, *p* = 0.001) (Table 3).

**Table 3:** Chi-Square analysis of ultrasound findings, multivariate logistic regression predictors and ROC analysis; AUC values for individual and combined ultrasound predictors of high-grade VUR (*N* = 406).

Ultrasound Variable	High-Grade VUR *n* = 50 (%)	No HG-VUR *n* = 356 (%)	*χ*^2^ Value	df	*p*-value
Hydronephrosis					
Right ((Grade ≥3)	18 (36.0%)	40 (11.2%)	19.982	1	<0.001**
Left (Grade ≥3)	25 (50.0%)	68 (19.1%)	21.986	1	<0.001**
Ureteral Dilatation					
Right Side	14 (28.0%)	26 (7.3%)	18.879	1	<0.001**
Left Side	16 (32.0%)	29 (8.1%)	22.951	1	<0.001**
Cortical Thinning					
Right Side	8 (16.0%)	19 (5.3%)	6.404	1	0.011*
Left Side	7 (14.0%)	24 (6.7%)	2.327	1	0.127
Renal Scarring					
Right Side	6 (12.0%)	13 (3.7%)	5.106	1	0.024*
Left Side	4 (8.0%)	11 (3.1%)	1.751	1	0.186
Bladder Abnormalities					
Wall Thickening	23 (46.0%)	131 (36.8%)	1.210	1	0.271
Distention	45 (90.0%)	238 (66.9%)	10.054	1	0.002**

Variable	B	OR	95% CI (Lower)	95% CI (Upper)	*p*-value
Right Hydronephrosis (Grade ≥3)	1.431	4.181	1.599	10.933	0.004**
Left Hydronephrosis (Grade ≥3)	1.512	4.538	2.158	9.541	<0.001**
Right Ureteral Dilatation	0.770	2.160	0.736	6.344	0.161
Left Ureteral Dilatation	0.501	1.651	0.645	4.224	0.296
Right Cortical Thinning	−0.594	0.552	0.153	1.990	0.364
Right Renal Scarring	1.324	3.758	1.084	13.033	0.037*
Bladder Distention (US)	1.431	4.183	1.533	11.415	0.005**
Model Fit Statistics					
Overall Model χ^2^	61.47	df = 7	*p* < 0.001		
Nagelkerke R^2^	0.267				

Predictor Variable	AUC	95% CI	Interpretation
Right Hydronephrosis (≥Grade 3)	0.624	0.540–0.708	Fair discriminative ability
Left Hydronephrosis (≥Grade 3)	0.654	0.574–0.734	Fair discriminative ability
Right Ureteral Dilatation	0.603	0.513–0.693	Fair discriminative ability
Left Ureteral Dilatation	0.619	0.535–0.703	Fair discriminative ability
Right Cortical Thinning	0.553	0.459–0.647	Poor discriminative ability
Right Renal Scarring	0.542	0.440–0.644	Poor discriminative ability
Bladder Distention (US)	0.616	0.531–0.701	Fair discriminative ability
Combined Ultrasound Model	0.824	0.757–0.891	Good–Excellent discriminative ability

** *p* < 0.01.

* *p* < 0.05 (two-tailed).

HG-VUR, High-Grade Vesicoureteral Reflux (Grades IV–V). df, degrees of freedom. Note. AUC interpretation: 0.50–0.60 = Poor; 0.60–0.70 = Fair; 0.70–0.80 = Good; 0.80–0.90 = Excellent. 95% CIs are approximate based on logistic regression predicted probability and individual binary predictors.

Ureteral dilatation on the right was found in 14/50 patients with high-grade VUR only but in 26/356 non-high-grade VUR (28.0% and 7.3%, respectively). A chi-square value of 18.879 (df = 1, *p* < 0.001) was obtained, which confirms that there is a highly significant association (Table 3). The same occurred with left-sided ureteral dilatation, where 16 patients with high-grade VUR (32.0%) and 29 patients with non-high-grade VUR (8.1%) were found, and the chi-square value was 22.951 (*p* < 0.001) (Table 3). These outcomes reveal that bilateral ureteral dilatation is a significant predictor of high-grade VUR.

Cortical thinning on the right side was observed in 8 patients with high-grade VUR (16.0%) and 19 patients with non-high-grade VUR (5.3%), with a statistically significant result (*χ*^2^ = 6.404, df = 1, *p* = 0.011) (Table 3). This indicates that there is a significant correlation between right-sided cortical thinning and severe reflux, which is potentially reflux nephropathy. However, on the left side, cortical thinning was detected in 7 high-grade VUR individuals (14.0%) and 24 non-high-grade VUR individuals (6.7%), with no significant association between the two (*χ*^2^ = 2.327, *p* = 0.127) (Table 3).

Right-sided renal scarring was observed in 6 high-grade VUR (12.0%) and 13 patients without high-grade VUR (3.7%) with a statistically significant chi-square value of 5.106 (df = 1, *p* = 0.024). Left-sided renal scarring, however, was not statistically significant (*χ*^2^ = 1.751, *p* = 0.186) (Table 3).

Thickening of the bladder wall was noted in 23/50 high-grade VUR (46.%), and 131/356 non-high-grade VUR (36.8%) (Table 3). The chi-square test was not statistically significant (*χ*^2^ = 1.210, df = 1, *p* = 0.271) (Table 3). Conversely, bladder distention on ultrasound was significantly associated (*χ*^2^ = 10.054, df = 1, *p* = 0.002) with 45 out of 50 high-grade VUR patients (90.0%) demonstrating bladded distention against 238 out of 356 atypical grade VUR patients (66.9%) (Table 3).

### 3.5 Multivariate logistic regression analysis

Multivariate binary logistic regression was used to detect independent predictors of high-grade VUR, after adjustment for the other ultrasound variables. High-grade VUR (1 = present, 0 = absent) was the dependent variable, and right and left hydronephrosis (Grade 3 and above), right and left ureteral dilatation, right cortical thinning, right renal scar, and distention of the bladder were the independent variables.

The logistic regression total model was found to be significant (*χ*^2^ = 61.47, 7, *p* < 0.001) with a Nagelkerke R^2^ of 0.267 that demonstrated the group of ultrasound predictor variables collectively explain about 26.7% of the variance in the high-grade VUR status (Table 3). Four statistically significant independent predictors of high-grade VUR were identified. The strongest predictor was left hydronephrosis of Grade 3 or greater, with an odds ratio (OR) of 4.538 (95% CI: 2.15895.41) implying that patients with severe left-sided hydronephrosis have a high chance of high-grade VUR (4.5 times more than patients with no hydronephrosis). Right hydronephrosis (Grade ≥3) was also a significant independent predictor (OR = 4.181, 95% CI: 1.599–10.933, *p* = 0.004) (Table 3).

Ultrasound bladder distention was another predictor included in the model (OR = 4.183, 95% CI: 1.533–11.415, *p* = 0.005), indicating that the presence of a distended bladder during ultrasound is strongly independent of high-grade VUR (Table 3).

Another important predictor was right renal scarring (OR = 3.758, 95% CI: 1.08413.033, *p* = 0.037), which suggests that reported parenchymal scarring can be an indicator of high-grade reflux with renal injury (Table 3).

Ureteral dilatation on both sides and on the right side and cortical thinning on the right did not maintain significant effects in the multivariate model, indicating that their bivariate relationships might be confounded by hydronephrosis severity (Table 3).

### 3.6 ROC curve analysis and diagnostic accuracy of ultrasound findings of high-grade VUR

#### 3.6.1 Area under the curve (AUC) values for individual predictors

Individual ultrasound predictors showed the most significant individual AUCs of left-sided severe hydronephrosis (0.654), left ureteral dilatation (0.619), bladder distention (0.616), and right hydronephrosis (0.624) **(**Table 3). Such values are in the range of fair discriminative ability, which proves that no individual ultrasound result is adequate in determining the presence of high-grade or low-grade VUR. This aligns with the existing evidence that indicates ultrasound findings, alone, are not that efficient in predicting VUR severity. Nonetheless, with the combination of all significant ultrasound predictors to form a multivariate model, the AUC increased significantly to 0.824 (95% CI: 0.757 −0.891) (Table 3), which corresponds to excellent discriminating ability. This result shows the evident benefit of a unified, multi-parametric ultrasound system as opposed to basing on a specific imaging parameter.

**Figure 1:**
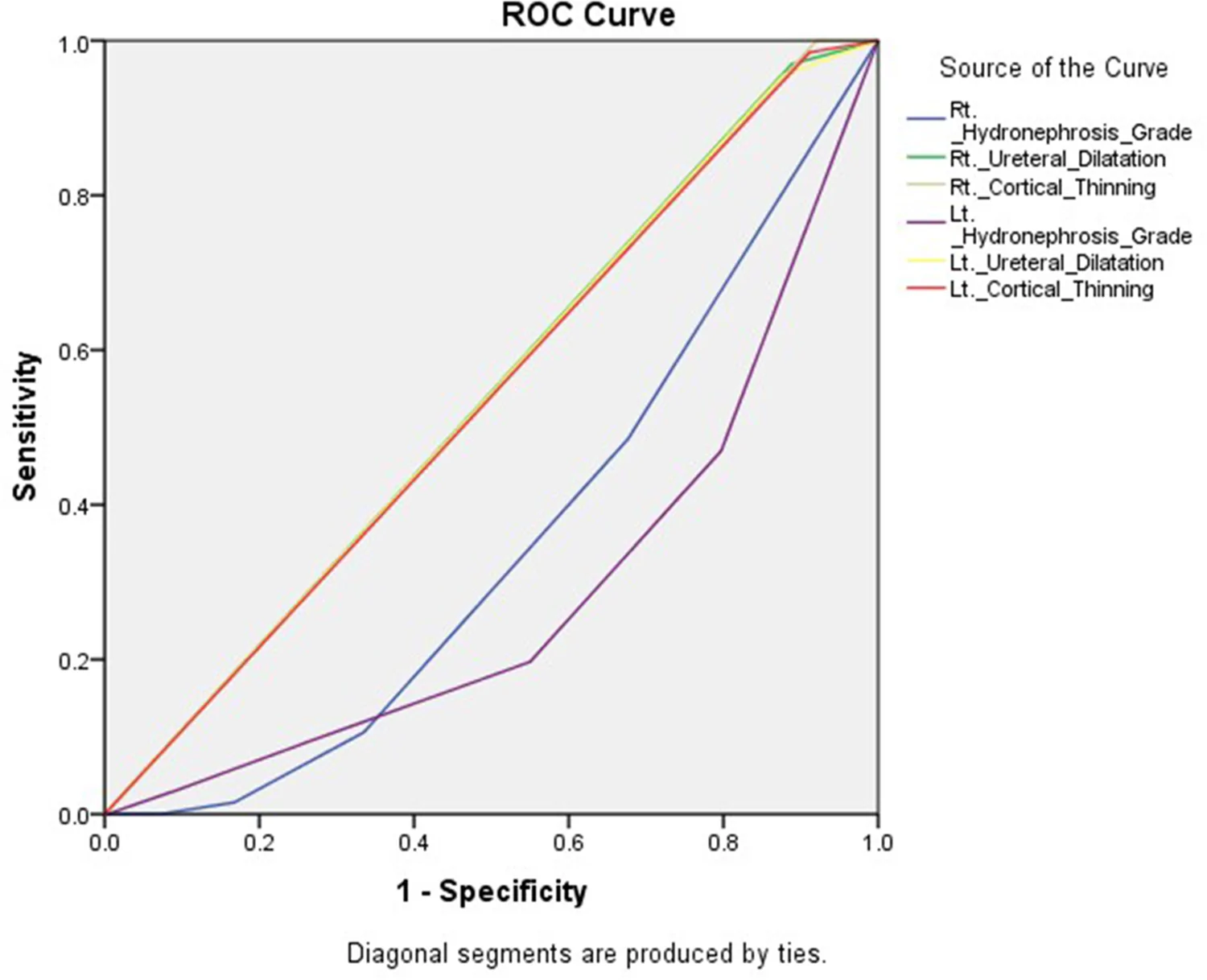
ROC curve: combined ultrasound predictive model for high-grade VUR. Area Under the Curve (AUC) = 0.824 | 95% CI: 0.757–0.891 | Optimal Cutoff (Youden Index) = 0.123, Sensitivity = 78.0% | Specificity = 76.4% at Optimal Cutoff, Reference line (AUC = 0.500) represents no discrimination (chance alone).

Figure 1 illustrates the ROC curve of the integrated ultrasound predictive model. Its AUC of 0.824 implies that the model is significantly above that of chance (AUC = 0.50). The Youden Index was then used to determine the best operating point on the curve, leading to the determination of the best cutoff probability of 0.123, where the model has a sensitivity of 78.0% and a specificity of 76.4%. This is the point based on which the sensitivity and specificity are maximised, and thus it is the most balanced point clinically regarding distinguishing between high-grade VUR patients and patients with no significant reflux. Diagnostic Accuracy of High-Grade VUR using VCUG as the Gold Standard.

The diagnostic accuracy of the combined ultrasound model as compared to VCUG as a reference was determined. Table 4 in the 2 × 2 contingency table reveals the frequency of true positives (TP), false positives (FP), false negatives (FN), and true negative negatives (TN) at the optimal cutoff point.

**Table 4:** A 2 × 2 contingency table of ultrasound model vs. VCUG Gold Standard (*N* = 406) and Summary of Diagnostic Performance Metrics for Combined Ultrasound Model for the detection of High-Grade VUR.

	VCUG: High-Grade VUR (Positive)	VCUG: No High-Grade VUR (Negative)	Total
Ultrasound: Positive	39 (TP)	84 (FP)	123
Ultrasound: Negative	11 (FN)	272 (TN)	283
Total	50	356	406

Metric	Value (%)	95% CI	Interpretation
Sensitivity	78.0%	66.3%–89.7%	Acceptable
Specificity	76.4%	71.8%–81.0%	Acceptable
Positive Predictive Value (PPV)	31.7%	23.3%–40.1%	Moderate
Negative Predictive Value (NPV)	96.1%	93.2%–99.0%	Excellent
Accuracy	76.6%		
Cohen's Kappa (*κ*)	0.335		Fair agreement

TP, True Positive; FP, False Positive; FN, False Negative; TN, True Negative. Based on optimal cutoff probability of 0.123 derived from Youden Index. Note. Metrics calculated at optimal cutoff (0.123). CI, Confidence Interval. PPV and NPV are influenced by the prevalence of high-grade VUR (12.3%) in this cohort.

The ultrasound model demonstrated good overall diagnostic performance for identifying high-grade VUR when compared with VCUG as the reference standard. Of the 50 patients with high-grade VUR confirmed on VCUG, ultrasound correctly identified 39 cases, resulting in a sensitivity of 78.0%. Among the 356 patients without high-grade VUR, ultrasound correctly classified 272 patients as negative, corresponding to a specificity of 76.4% (Table 4).

The positive predictive value of ultrasound was 31.7%, indicating that approximately one-third of patients with a positive ultrasound finding actually had high-grade VUR on VCUG. In contrast, the negative predictive value was 96.1%, demonstrating that a negative ultrasound result was highly reliable for excluding high-grade VUR. The overall diagnostic accuracy of ultrasound was 76.6%, with 311 of the 406 cases correctly classified (Table 4).

Assessment of agreement between ultrasound and VCUG yielded a Cohen's kappa coefficient of 0.335, indicating fair agreement beyond chance (Table 4). These findings suggest that although ultrasound has limited ability to confirm the presence of high-grade VUR because of its relatively low positive predictive value and modest agreement with VCUG, it performs well as a screening or exclusion tool due to its high negative predictive value. Consequently, a negative ultrasound examination substantially reduces the likelihood of high-grade VUR, whereas positive ultrasound findings may require confirmation with VCUG.

The figures of the above findings can be found in Supplementary File S1.

## 4 Discussion

This retrospective cross-sectional study evaluated the predictive value of renal and bladder ultrasound findings for the detection of high-grade vesicoureteral reflux (VUR) in a cohort of 406 pediatric patients from the Central Region of Saudi Arabia. The findings demonstrate that although individual ultrasound abnormalities possess only modest discriminatory ability for identifying severe reflux, a combined ultrasound model incorporating multiple sonographic parameters achieved good diagnostic performance with an area under the curve (AUC) of 0.824, sensitivity of 78.0%, specificity of 76.4%, and overall accuracy of 76.6%. Furthermore, the model exhibited an excellent negative predictive value (96.1%), suggesting an important clinical role for ultrasound in excluding high-grade VUR and reducing unnecessary invasive investigations.

The prevalence of high-grade VUR in this study was 12.3%, which is consistent with previously reported rates among children referred for urinary tract imaging because of antenatal hydronephrosis, recurrent urinary tract infections, or suspected congenital urinary tract abnormalities. VUR prevalence varies considerably depending on the indication for imaging, ranging from 10% to 40% in children investigated following febrile urinary tract infections and reaching even higher proportions among those with antenatal hydronephrosis or established renal abnormalities. Severe reflux remains clinically significant because of its association with recurrent pyelonephritis, renal scarring, hypertension, and chronic kidney disease later in life (11). Recent evidence continues to demonstrate that high-grade reflux is strongly associated with deterioration of renal function and reduction in renal size, emphasizing the importance of early diagnosis and risk stratification (12, 13).

The demographic characteristics of the present cohort are consistent with the epidemiology of pediatric urinary tract abnormalities. Nearly two-thirds of the study population were male and more than 60% were younger than 12 months of age. This predominance of infants reflects contemporary referral patterns following antenatal ultrasonographic detection of hydronephrosis and is consistent with international recommendations advocating early postnatal evaluation of urinary tract abnormalities. Antenatal hydronephrosis represented the commonest indication for imaging in this study, accounting for 45.3% of referrals. This finding supports the increasingly recognized role of prenatal screening in identifying children at risk of clinically significant reflux before the occurrence of urinary tract infections or irreversible renal injury.

One of the key findings of this study was the strong association between severe hydronephrosis and high-grade VUR. Both right-sided and left-sided hydronephrosis of grade 3 or higher were significantly associated with severe reflux in univariate analysis and remained independent predictors following multivariate adjustment. Children with severe left hydronephrosis had more than four times the odds of high-grade reflux compared with those without hydronephrosis, while severe right hydronephrosis demonstrated a similarly strong effect size.

These findings are biologically plausible, as persistent retrograde urine flow increases hydrostatic pressure within the collecting system, leading to progressive pelvicalyceal dilatation. The greater predictive value of left-sided hydronephrosis observed in the present study may reflect the higher baseline severity of hydronephrosis identified on the left side within this cohort, although the exact mechanism remains uncertain. Similar observations have been reported in recent studies demonstrating that increasing severity of hydronephrosis correlates strongly with reflux grade and adverse renal outcomes. Öztürk and colleagues identified severe hydronephrosis and distal ureteric enlargement as major ultrasound indicators of clinically significant reflux and treatment failure in pediatric VUR populations (2). Likewise, investigators evaluating antenatal hydronephrosis have shown that moderate-to-severe postnatal hydronephrosis substantially increases the probability of high-grade VUR and reflux nephropathy (14).

Ureteral dilatation also demonstrated a significant association with severe reflux during bivariate analysis. The prevalence of ureteral dilatation among patients with high-grade VUR was approximately four times greater than among patients without severe reflux. The finding is consistent with the pathophysiological progression of reflux disease in which elevated reflux pressures lead to progressive ureteral enlargement and tortuosity. However, after adjustment for hydronephrosis severity, ureteral dilatation no longer remained independently predictive in the multivariate model.

The loss of significance following adjustment suggests that ureteral dilatation may represent an intermediate manifestation of reflux severity rather than an independent pathological process. Hydronephrosis and ureteral dilatation frequently coexist and are likely to share a substantial proportion of diagnostic information. Similar findings have been reported in other studies in which ureteral diameter contributed significantly to prediction models but demonstrated reduced independent value once hydronephrosis severity was included. Therefore, ureteral dilatation should be interpreted within the broader context of collecting system abnormalities rather than as an isolated predictor.

Cortical thinning and renal scarring represent indicators of chronic renal injury and reflux nephropathy rather than early manifestations of reflux itself. Right-sided cortical thinning demonstrated a statistically significant association with high-grade VUR in univariate analysis, whereas left-sided cortical thinning did not achieve significance. Similarly, right-sided renal scarring was significantly associated with severe reflux and remained an independent predictor in multivariate analysis with an odds ratio approaching four.

These findings support existing evidence linking renal parenchymal damage with persistent or recurrent severe reflux. Renal scarring develops through repeated episodes of pyelonephritis and sustained intrarenal pressure changes associated with severe VUR. The identification of renal scarring on ultrasound should therefore raise immediate clinical suspicion regarding underlying high-grade reflux and potential long-term renal dysfunction. Recent pediatric studies continue to emphasize the association between high-grade VUR, intrarenal reflux, renal size reduction, and declining renal function, reinforcing the importance of early identification before irreversible injury occurs (12).

An unexpected finding of this study was the significant independent predictive value of bladder distention on ultrasound. Patients with bladder distention had more than fourfold higher odds of high-grade VUR after adjustment for other imaging variables. However, this finding should be interpreted with caution. In pediatric ultrasound, bladder distention may simply reflect physiological bladder filling resulting from hydration or the timing of the examination rather than pathological bladder dysfunction. Because this was a retrospective study, standardized bladder filling protocols and clinical data regarding bladder bowel dysfunction (BBD), neurogenic bladder, voiding symptoms, and post-void residual urine were not consistently available and therefore could not be evaluated. Consequently, bladder distention in the present study should be regarded as a surrogate imaging marker associated with high-grade VUR rather than direct evidence of underlying functional bladder abnormalities. Nevertheless, abnormal bladder dynamics, including BBD and neurogenic bladder, are well-recognized risk factors for persistent VUR and recurrent urinary tract infection. Future prospective studies integrating standardized ultrasound protocols with comprehensive clinical assessment of bladder function are required to determine whether bladder distention represents an independent biological predictor of severe reflux or is partly attributable to physiological bladder filling during imaging.

Bladder wall thickening, however, did not demonstrate a significant relationship with high-grade reflux in the current study. This finding differs from some previous reports that have associated bladder wall abnormalities with recurrent infection and dysfunctional voiding. Nevertheless, bladder wall thickness measurements in pediatric populations are highly dependent on bladder filling volume and imaging technique, potentially explaining the lack of association observed in this cohort.

ROC analysis demonstrated the added value of the combined ultrasound model. Individual ultrasound findings demonstrated AUC values ranging from 0.542 to 0.654, indicating poor to fair discrimination. No single ultrasound parameter was sufficiently accurate to replace VCUG as a standalone diagnostic test for high-grade reflux. This observation is consistent with previous studies demonstrating the limitations of isolated ultrasound findings in predicting VUR severity.

The performance of the combined ultrasound model was substantially better, achieving an AUC of 0.824, which indicates good to excellent discriminative performance. These finding highlights the value of multiparametric imaging assessment rather than reliance on a single abnormality. The integration of hydronephrosis severity, renal scarring, bladder abnormalities, and ureteric changes likely captures different stages of reflux pathophysiology and therefore improves predictive accuracy.

The diagnostic performance of the combined ultrasound model has important clinical implications for the evaluation of children with suspected VUR. The excellent NPV (96.1%) indicates that children classified as low risk by the ultrasound model are unlikely to have high-grade VUR, suggesting that ultrasound may serve as an effective screening or triage tool in selected clinical settings. In children with low clinical suspicion, a negative ultrasound assessment may help reduce the need for immediate VCUG, thereby minimizing exposure to ionizing radiation, procedural discomfort, and healthcare costs. However, VCUG remains the reference standard for the diagnosis and grading of VUR and should continue to be performed in children with recurrent febrile urinary tract infections, persistent clinical suspicion despite negative ultrasound findings, severe sonographic abnormalities, or other established high-risk features (11). Accordingly, the combined ultrasound model should be considered as a complementary risk-stratification tool rather than a replacement for VCUG, helping clinicians identify patients who are most likely to benefit from definitive fluoroscopic evaluation.

The prevalence of high-grade VUR in this cohort was only 12.3%, indicating that the high NPV value could be influenced by disease prevalence. Predictive values could differ in populations with a different prevalence of high-grade VUR, whereas sensitivity and specificity are generally more transportable across settings.

The high NPV implies that when the ultrasound model classifies a patient as negative, the probability of underlying high-grade reflux is extremely low. Clinically, this means that ultrasound may serve effectively as a screening or triage tool to identify children who may safely avoid immediate VCUG investigation. This approach aligns with modern pediatric imaging strategies that prioritize minimizing radiation exposure and reducing invasive procedures whenever possible.

Conversely, the positive predictive value of only 31.7% indicates that many patients with positive ultrasound findings did not ultimately have severe reflux on VCUG. This relatively low PPV is partly explained by the low prevalence of high-grade VUR in the study population because predictive values are heavily influenced by disease prevalence. Nevertheless, this finding reinforces the principle that abnormal ultrasound findings alone should not be considered diagnostic of severe reflux and require confirmation using definitive imaging techniques.

The Cohen's kappa coefficient of 0.335 demonstrated fair agreement between ultrasound and VCUG beyond chance alone. Although this level of agreement is insufficient to justify replacement of VCUG, it confirms meaningful concordance between the two modalities. Similar studies evaluating conventional renal ultrasound have consistently demonstrated only fair-to-moderate agreement with VCUG findings, supporting the conclusion that ultrasound should complement rather than replace standard reflux imaging protocols.

These findings should also be interpreted within the context of rapidly evolving imaging technologies. ceVUS has emerged as a highly sensitive, radiation-free alternative to VCUG for the diagnosis and grading of VUR (15). Multiple studies have demonstrated diagnostic performance comparable to conventional VCUG while avoiding ionizing radiation exposure and reducing patient discomfort. Recent comparisons between ceVUS and VCUG continue to show excellent concordance between both techniques, particularly for clinically significant reflux grades (16, 17).

Similarly, advances in contrast-enhanced ultrasound and elastographic assessment are improving the ability to detect early reflux nephropathy and renal parenchymal injury before irreversible scarring occurs. These technologies may eventually bridge the diagnostic gap between conventional ultrasound and invasive fluoroscopic investigations.

Artificial intelligence and machine learning approaches are also likely to transform pediatric reflux imaging over the coming decade. Emerging predictive algorithms integrating ultrasound features, clinical variables, and demographic characteristics have demonstrated promising accuracy in identifying severe reflux while reducing unnecessary VCUG examinations. Recent machine learning studies have reported excellent predictive performance and objective grading of reflux severity, suggesting that future diagnostic pathways may increasingly incorporate automated risk stratification models (18).

From a healthcare systems perspective, the findings of this study have particular relevance for Saudi Arabia and other countries pursuing healthcare modernization initiatives. Reducing unnecessary VCUG examinations would lower healthcare expenditure, decrease procedural burden, and minimize radiation exposure among children. Such an approach aligns closely with national healthcare transformation goals emphasizing precision medicine, digital health, and evidence-based resource utilization and management of VCUR (19).

Several limitations should be acknowledged when interpreting these findings. First, the retrospective design introduces the possibility of selection bias and incomplete clinical documentation since only children who underwent both ultrasound and VCUG were included, the study population may represent a selected cohort with a relatively high pre-test probability of VUR. Second, the study relied on previously reported ultrasound examinations rather than centralized image re-evaluation, which may introduce interobserver variability. Third, because the study was conducted in referral hospitals within a single geographic region, the findings may not be fully generalizable to all pediatric populations. Forth, standardized bladder filling protocols and detailed clinical information on BBD, neurogenic bladder, voiding symptoms, and post-void residual urine were unavailable because of the retrospective study design. Therefore, the observed association between bladder distention and high-grade VUR may have been influenced by physiological bladder filling or residual confounding and should be interpreted cautiously. Fifth, the high NPV is influenced by the relatively low prevalence of high-grade VUR in this cohort, and the predictive values may differ in populations with different disease prevalence. Lastly, the model's PPV remains limited. Therefore, a positive ultrasound-based model should not be interpreted as confirmatory for high-grade VUR.

Despite these limitations, the study possesses several important strengths, including a relatively large sample size, use of VCUG as the gold standard reference, inclusion of multiple ultrasound parameters, and evaluation of both individual and combined predictive performance. To our knowledge, this represents one of the largest contemporary studies evaluating ultrasound predictors of severe VUR in Saudi children.

Although the proposed ultrasonographic prediction model demonstrated good discriminative ability in this retrospective cohort, further validation is required before its adoption into routine clinical practice. Prospective studies involving larger and more diverse patient populations, preferably across multiple institutions, are necessary to confirm the model's reproducibility, calibration, and generalizability. External validation will also help determine whether the identified predictors and proposed probability threshold remain applicable in different clinical settings. Such validation studies will be critical to establish the clinical utility of the model and define its role in guiding decisions regarding the selective use of VCUG in children at risk of high-grade VUR.

Future studies should focus on prospective multicenter validation of the predictive model in larger and more diverse populations. The incorporation of advanced imaging techniques such as ceVUS, Doppler ureteric jet analysis, elastography, and artificial intelligence algorithms may further improve non-invasive diagnosis and reduce reliance on VCUG.

## 5 Conclusion

In conclusion, ultrasound has substantial value as a non-invasive screening modality for identifying children at risk of high-grade vesicoureteral reflux. Severe hydronephrosis, renal scarring, and bladder distention were the strongest independent sonographic predictors of severe reflux. Although ultrasound cannot replace VCUG because of its limited positive predictive value and only fair agreement with the reference standard, its excellent negative predictive value makes it an effective exclusion tool. Future research should focus on prospective multicenter validation studies and integration of advanced ultrasound techniques and artificial intelligence-based prediction models to further refine non-invasive risk stratification strategies for pediatric VUR. The multiparametric ultrasound model may help identify children at low risk of high-grade VUR and support more selective use of VCUG, but it should not be seen as a replacement for VCUG.

## Data Availability

The original contributions presented in the study are included in the article/Supplementary Material, further inquiries can be directed to the corresponding author.
