# Supplementary material for: Predictive value of ultrasound findings for high-grade vesicoureteral reflux in children: a retrospective cross-sectional study in Saudi Arabia
Source: Front Pediatr. 2026 Aug 19;14:1932063. doi: 10.3389/fped.2026.1932063 (PMC13534030; doi:10.3389/fped.2026.1932063)
Supplement: Supplementary file 1 [file Datasheet1.pdf]

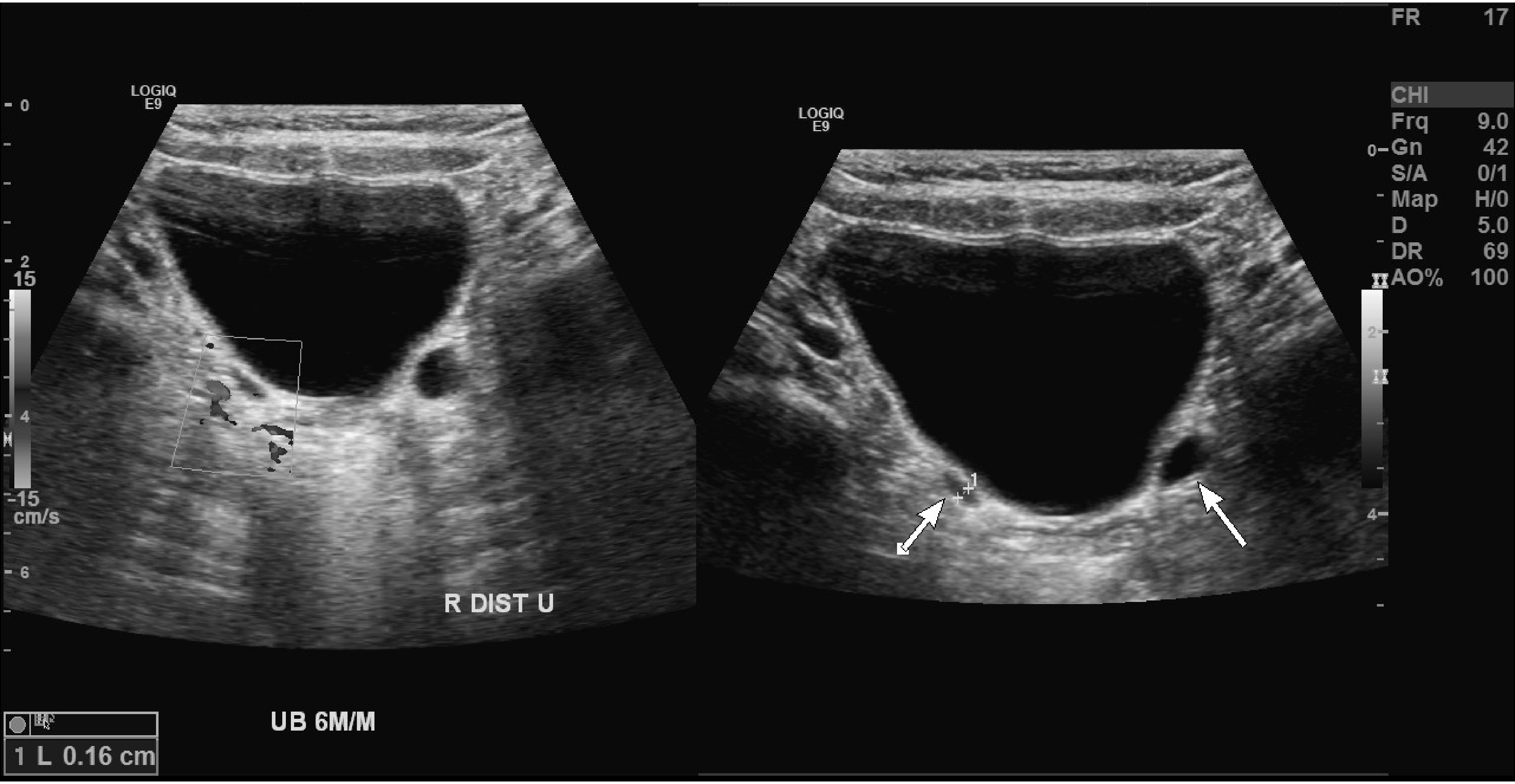
LOGIQ
E9
R DIST U
UB 6M/M
1 L 0.16 cm
15
-15
cm/s
LOGIQ
E9
FR 17
CHI
Frq 9.0
Gn 42
S/A 0/1
Map H/0
D 5.0
DR 69
AO% 100

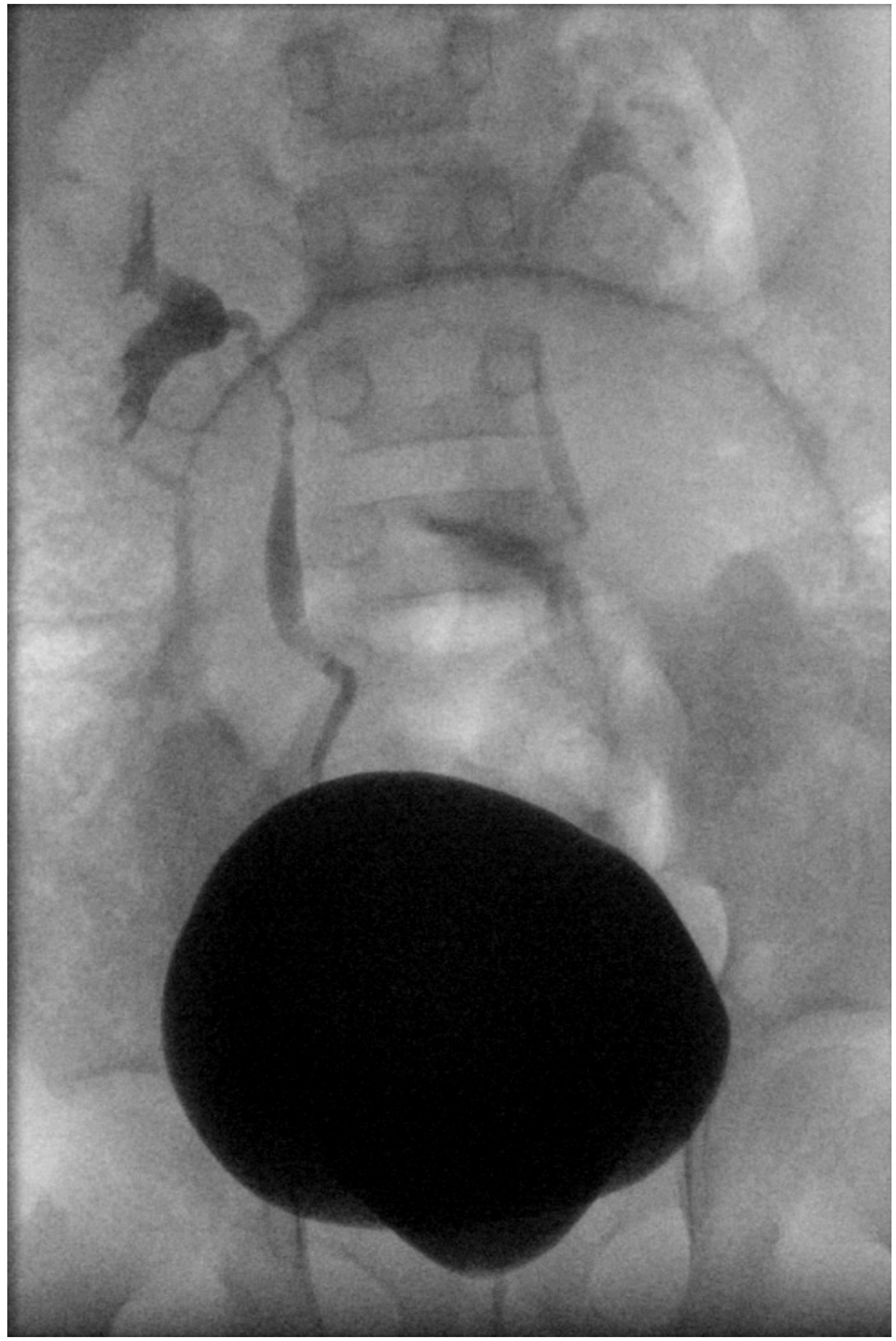

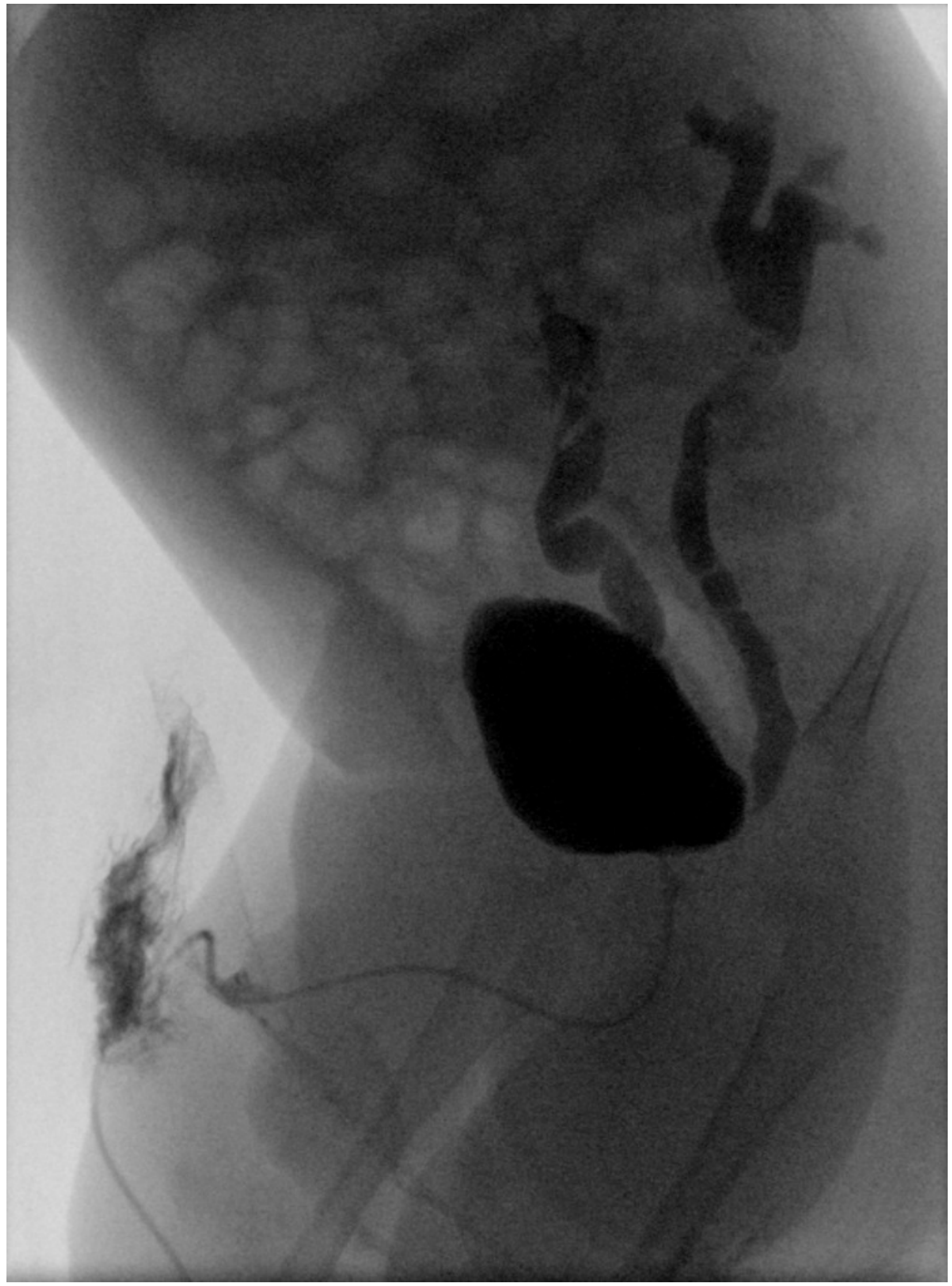

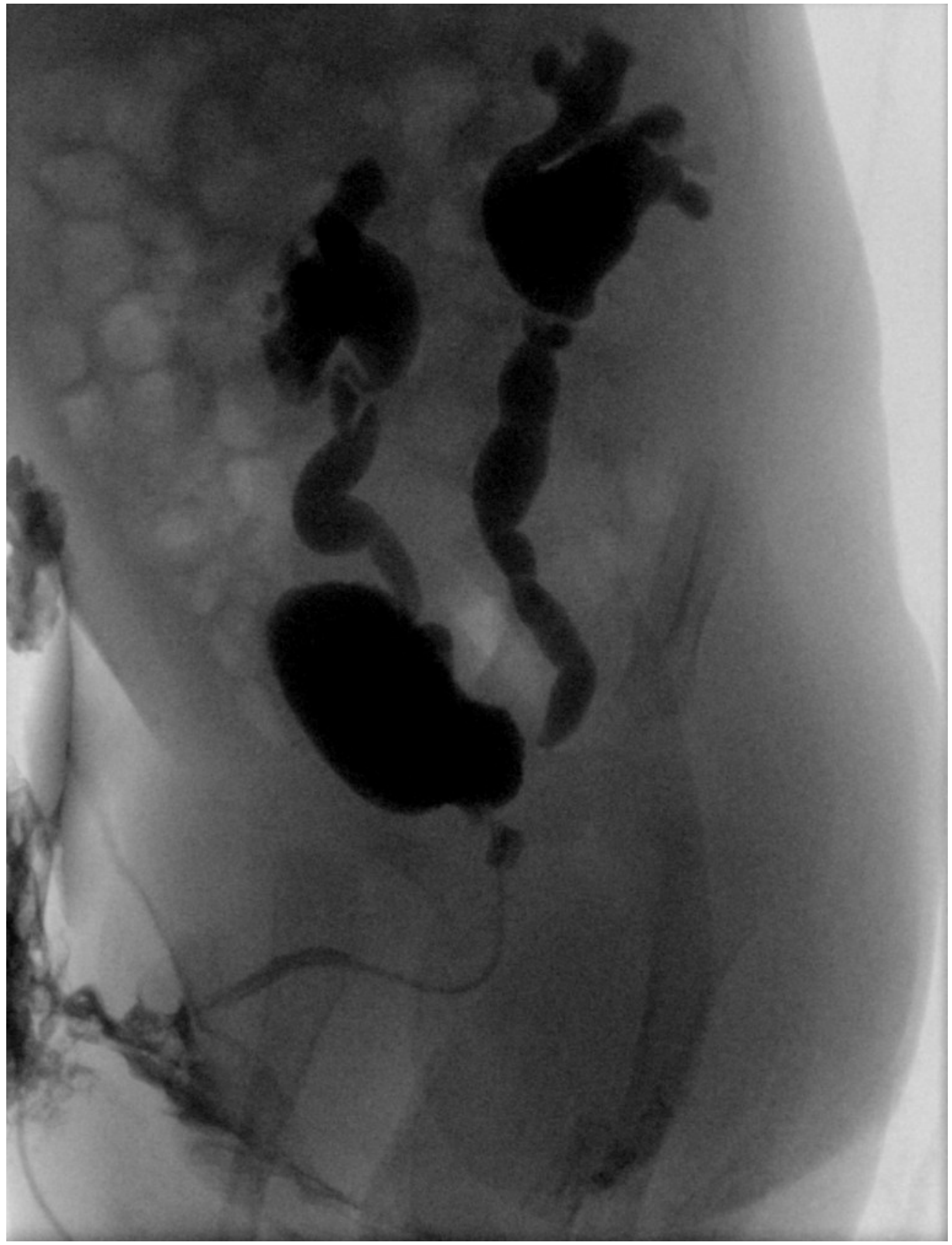

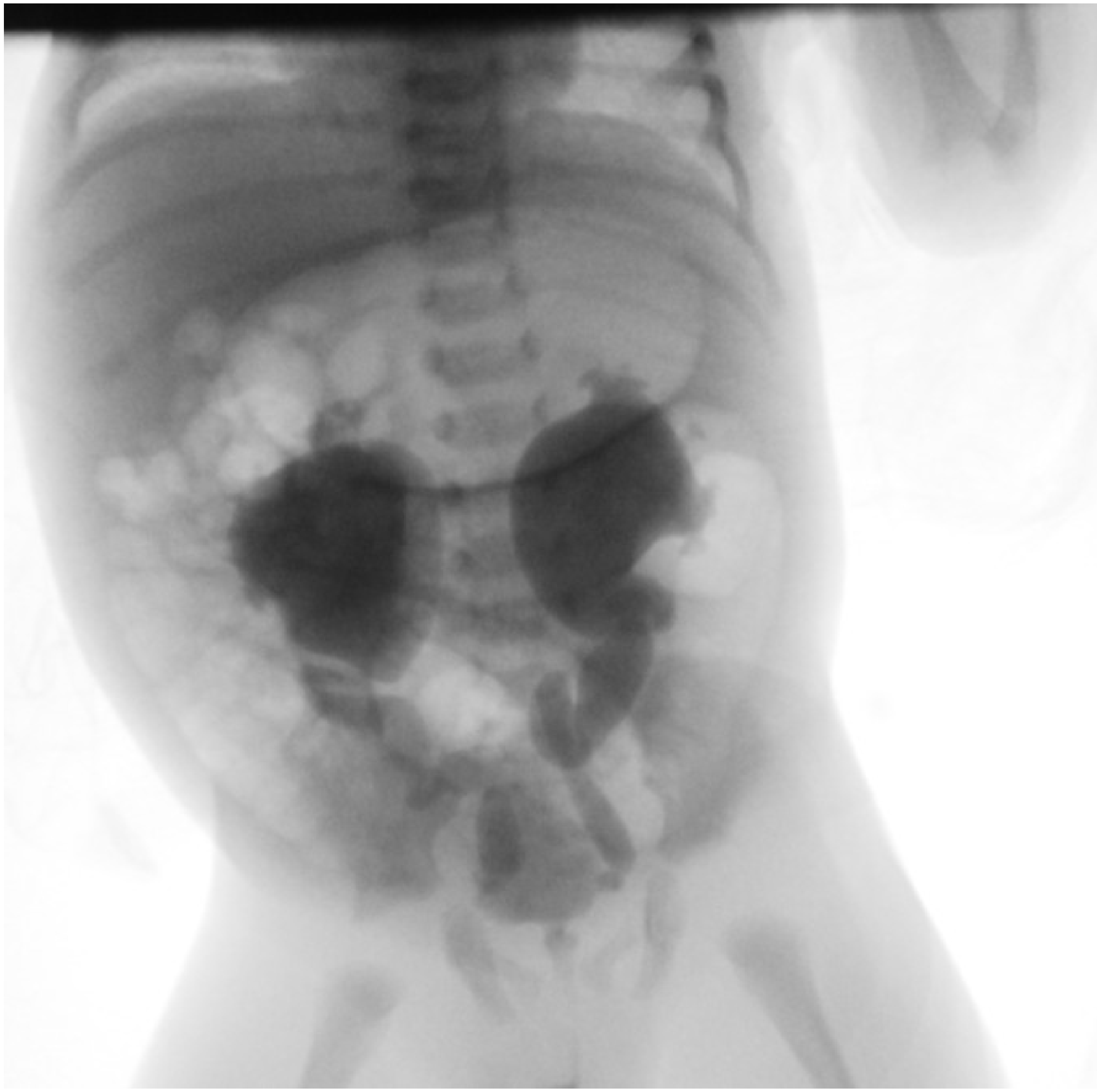

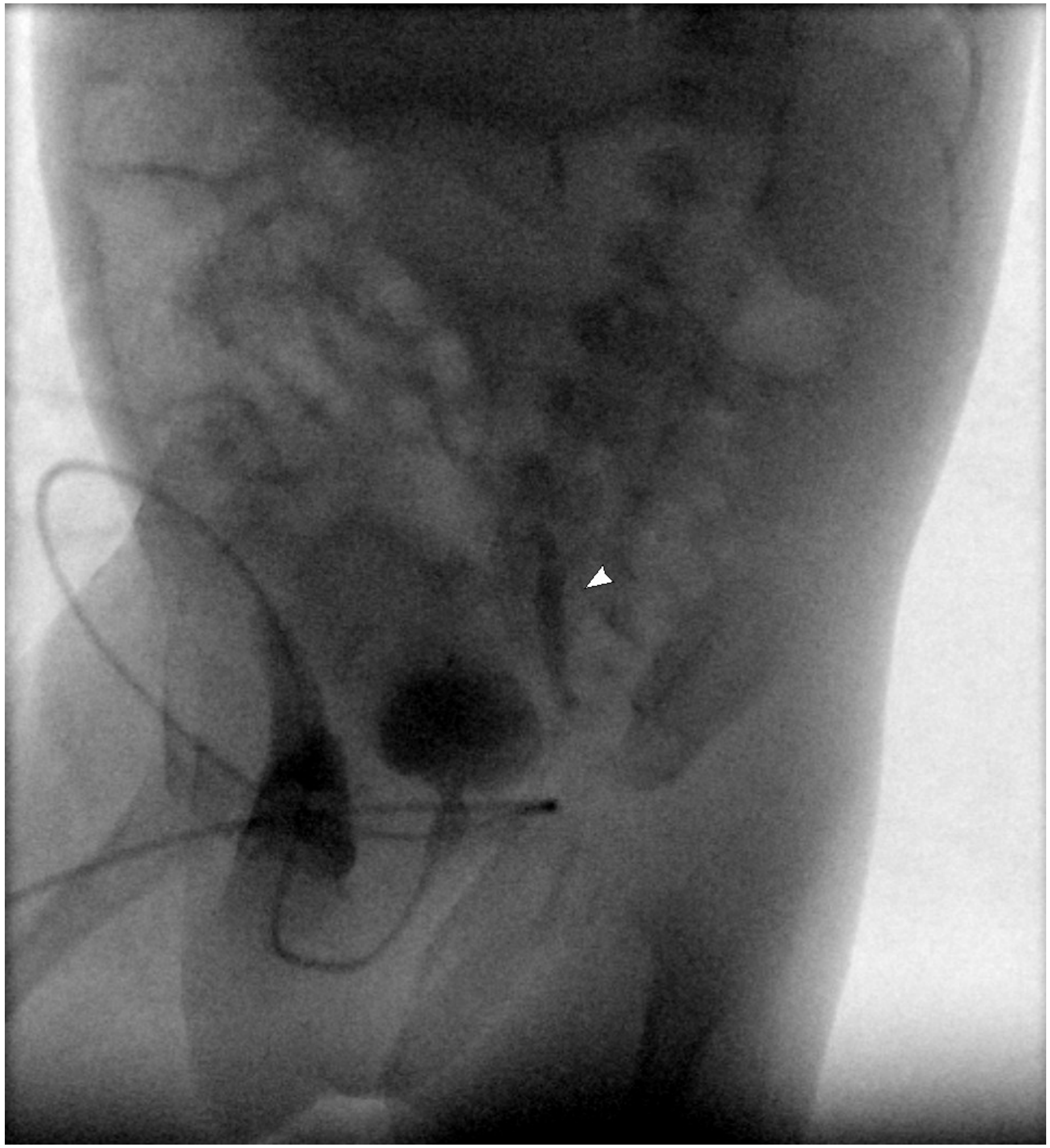

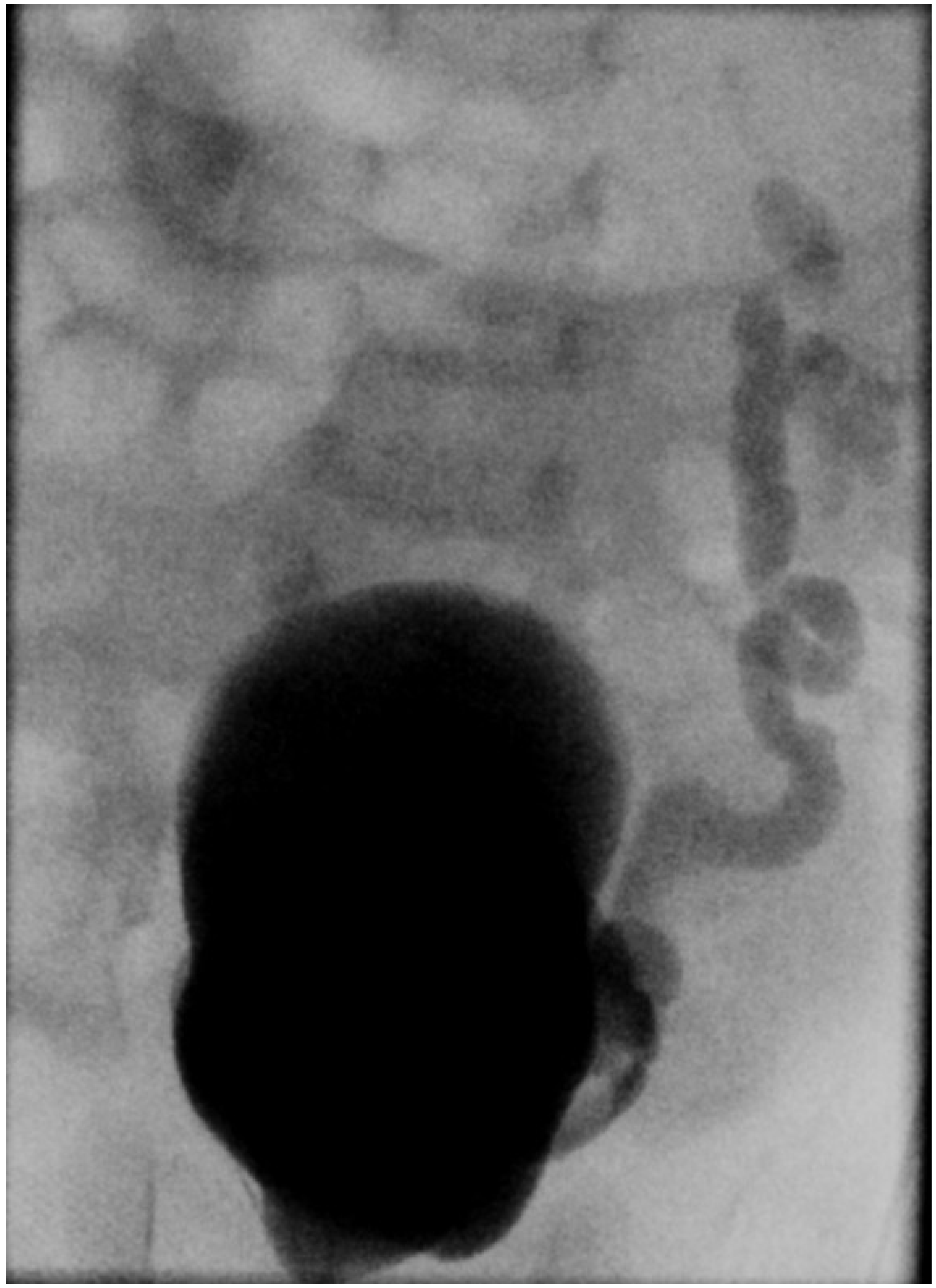

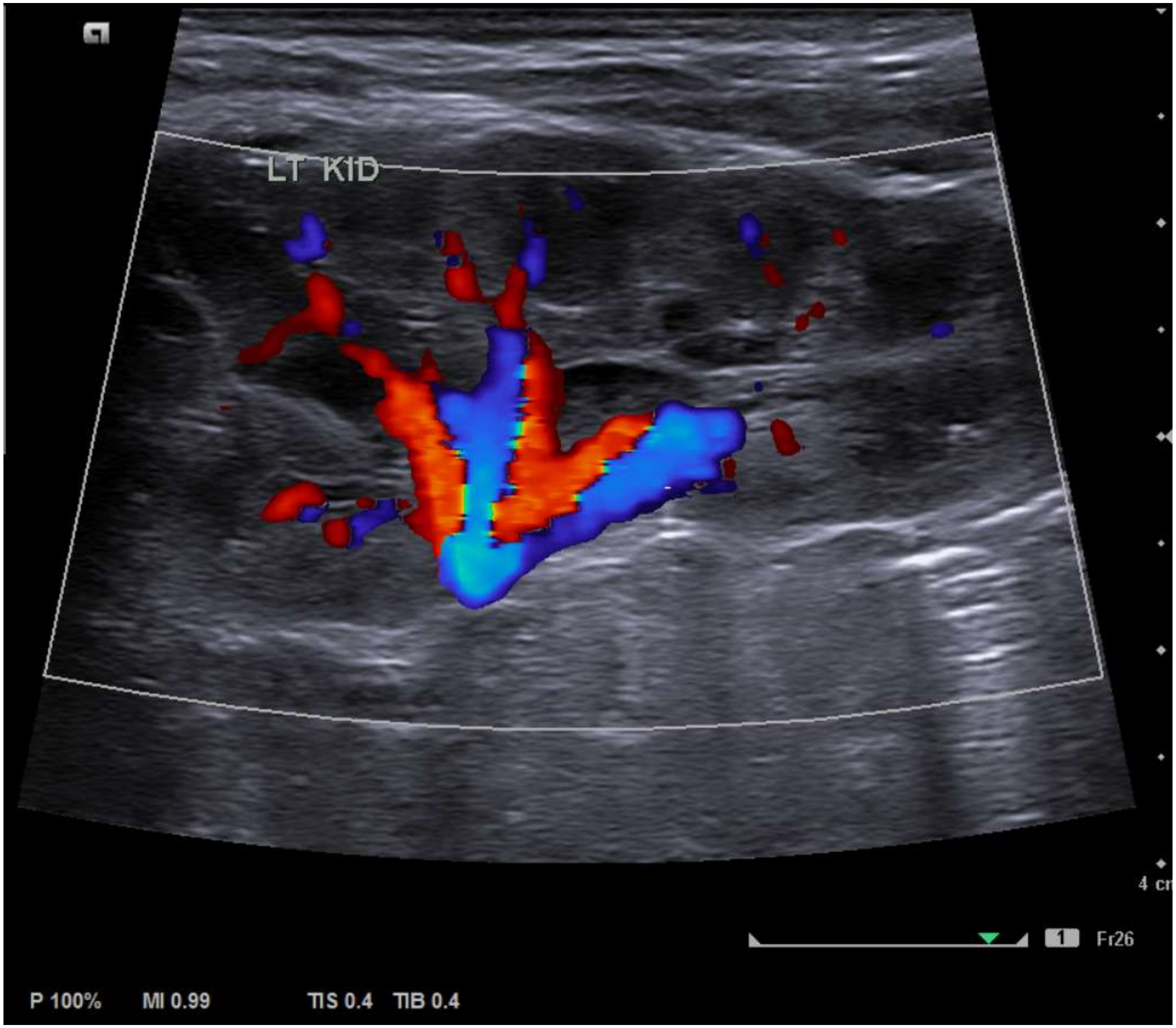
LT KID
4 cm
1
Fr26
P 100%
MI 0.99
TIS 0.4
TIB 0.4

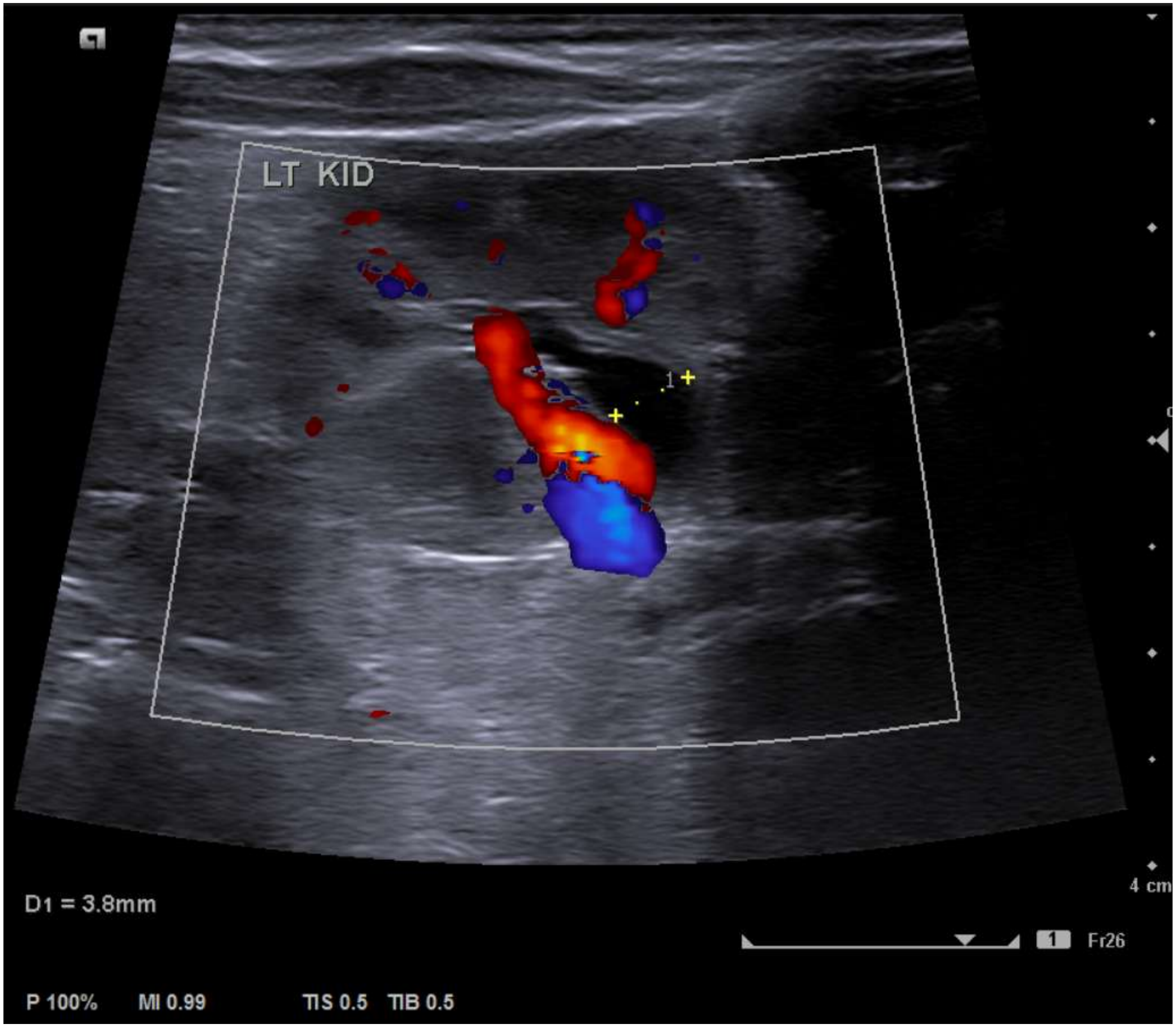
LT KID
1
4 cm
D1 = 3.8mm
1
Fr26
P 100%
MI 0.99
TIS 0.5
TIB 0.5

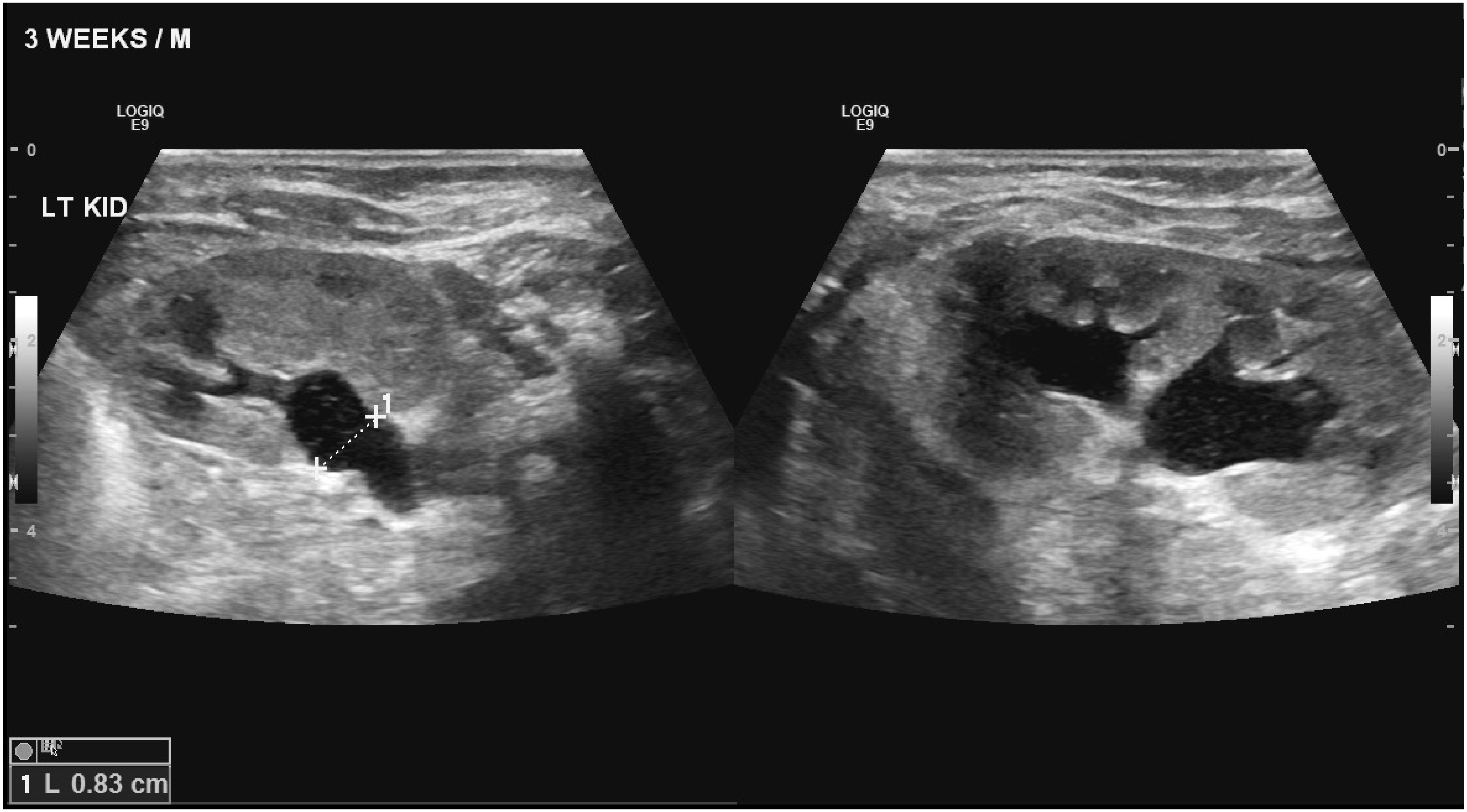

3 WEEKS / M
LOGIQ
E9
LT KID
LOGIQ
E9
1 L 0.83 cm

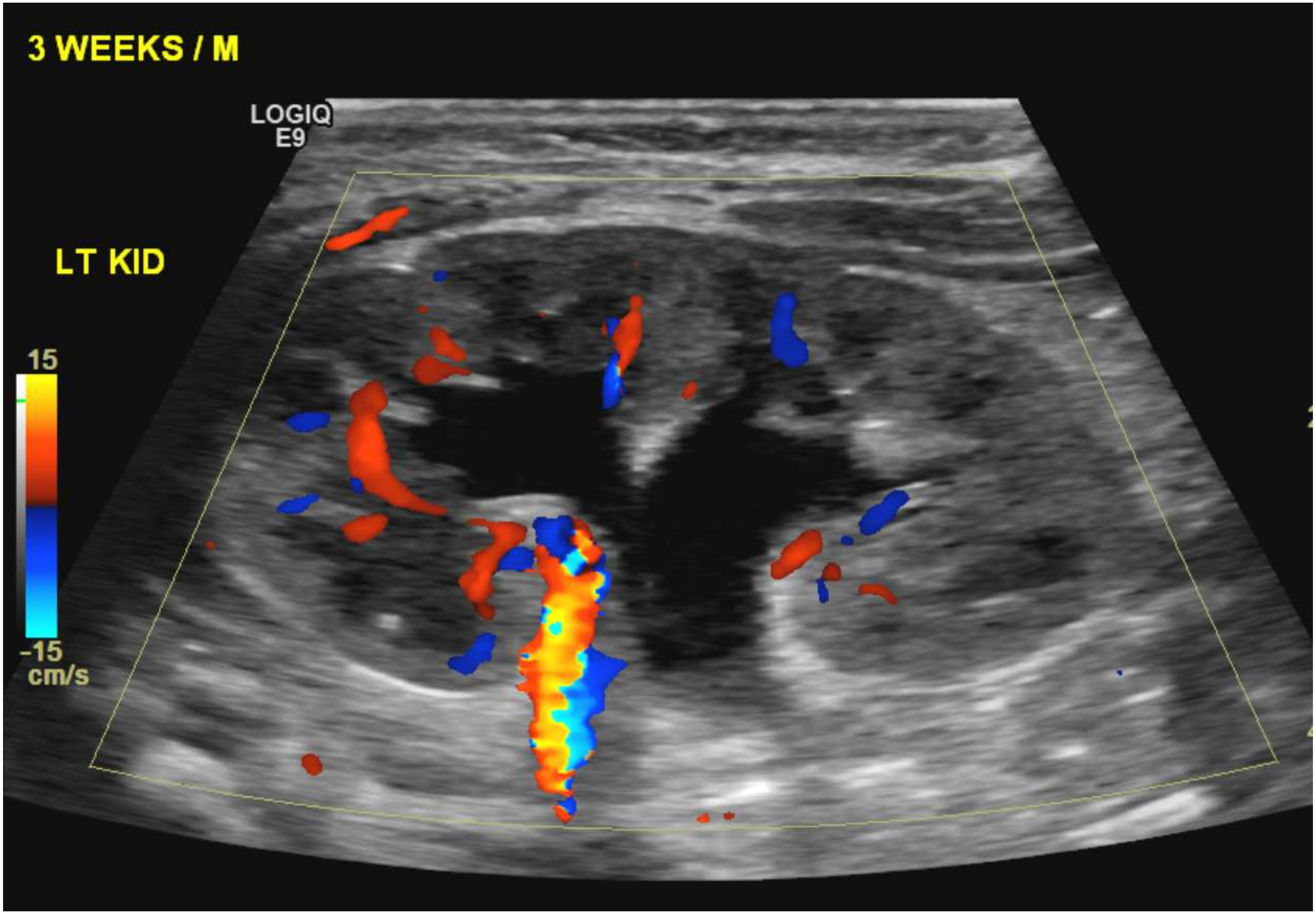
3 WEEKS / M
LOGIQ
E9
LT KID
15
-15
cm/s

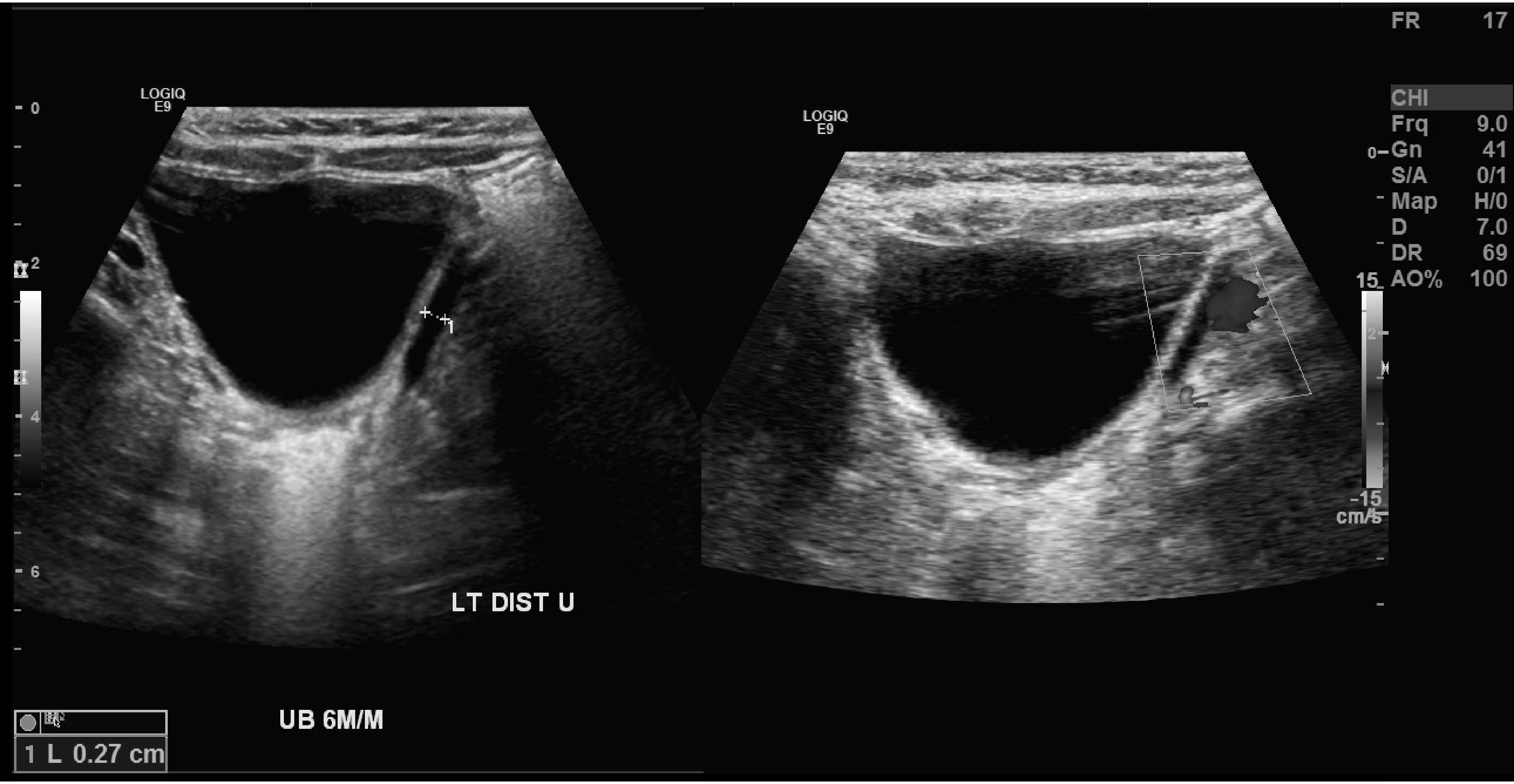

LOGIQ E9
LOGIQ E9
LT DIST U
UB 6M/M
1 L 0.27 cm
FR 17
CHI
Frq 9.0
Gn 41
S/A 0/1
Map H/0
D 7.0
DR 69
AO% 100
15
-15
cm/s

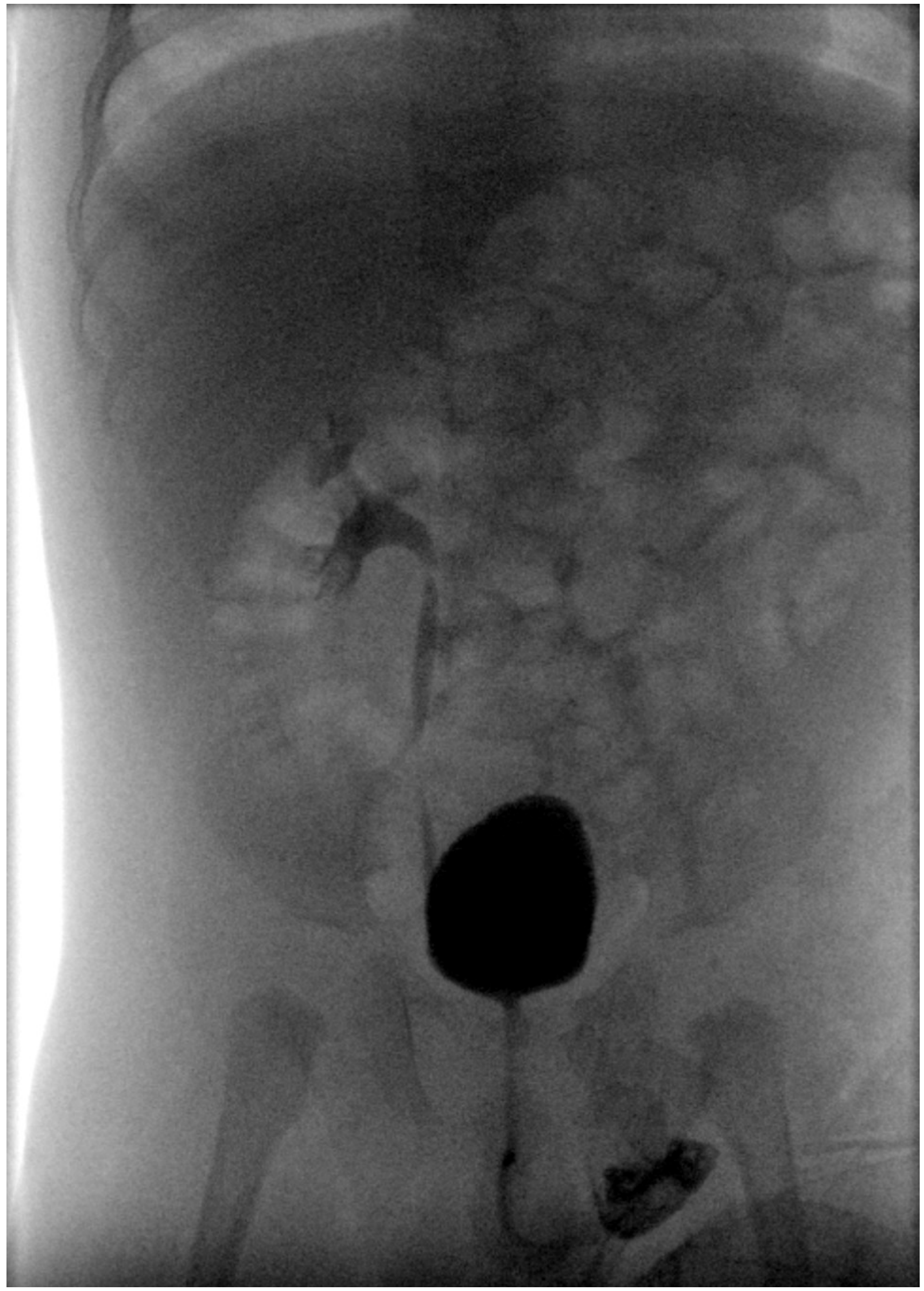

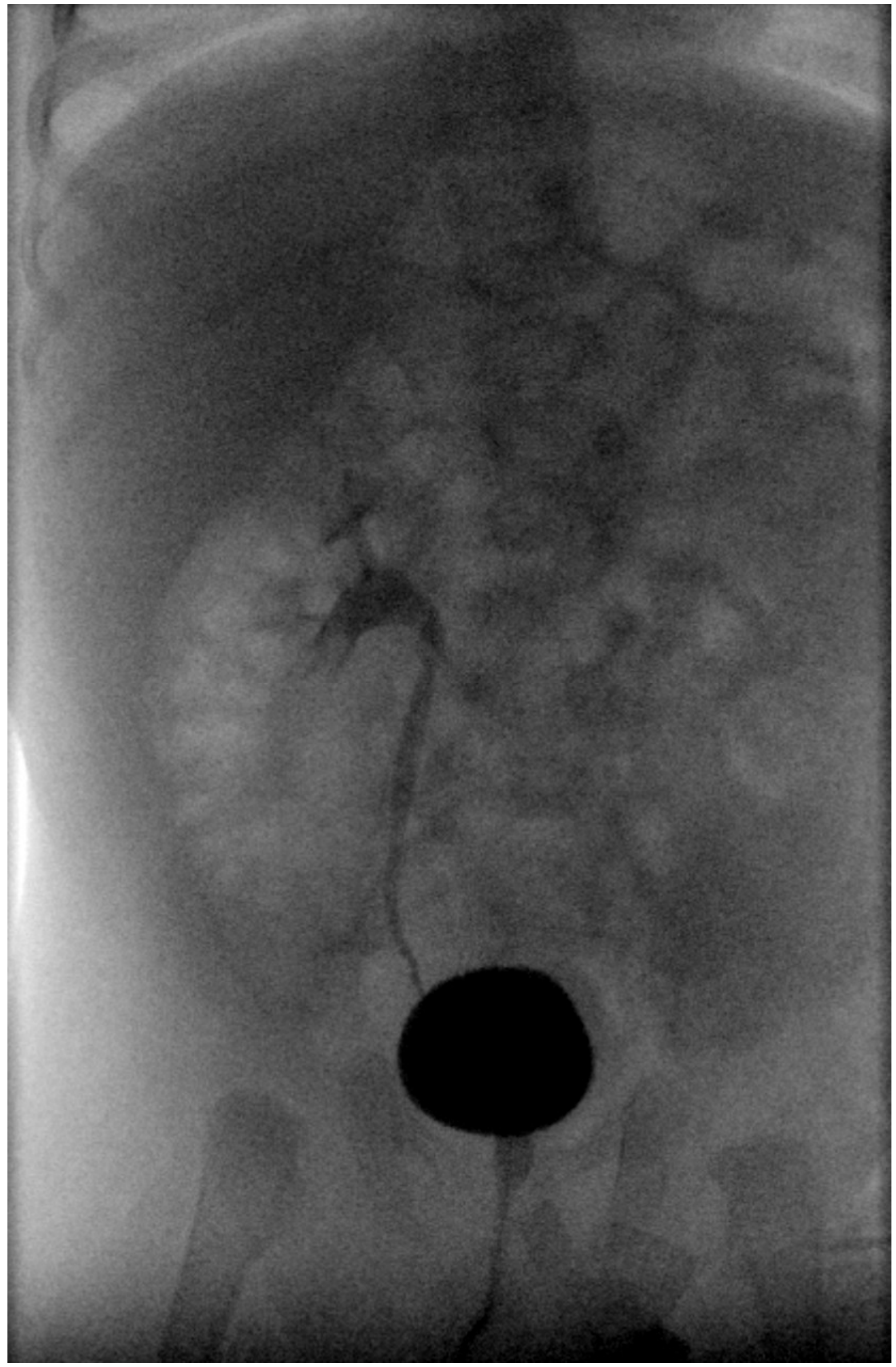

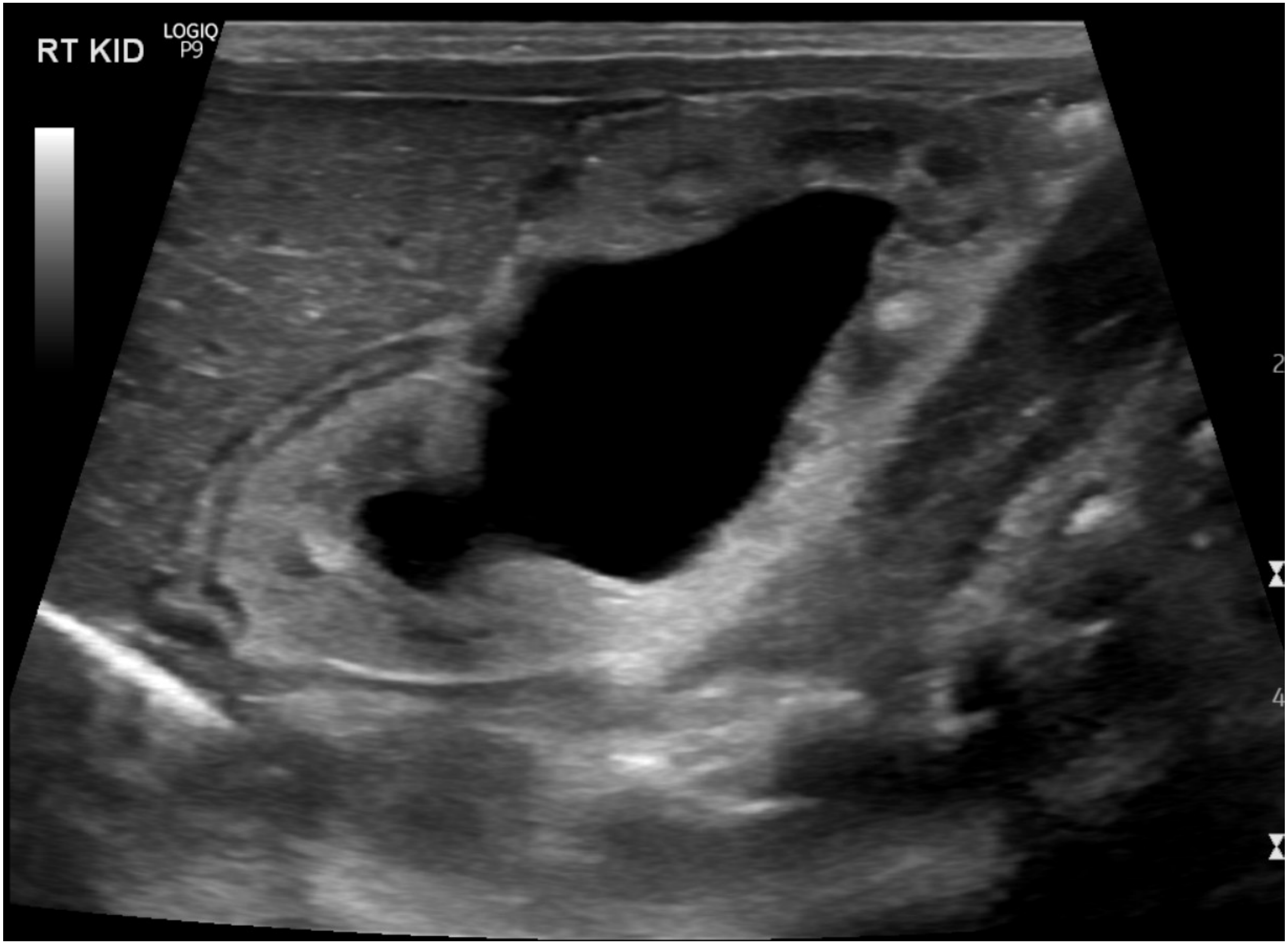
RT KID
LOGIQ
P9

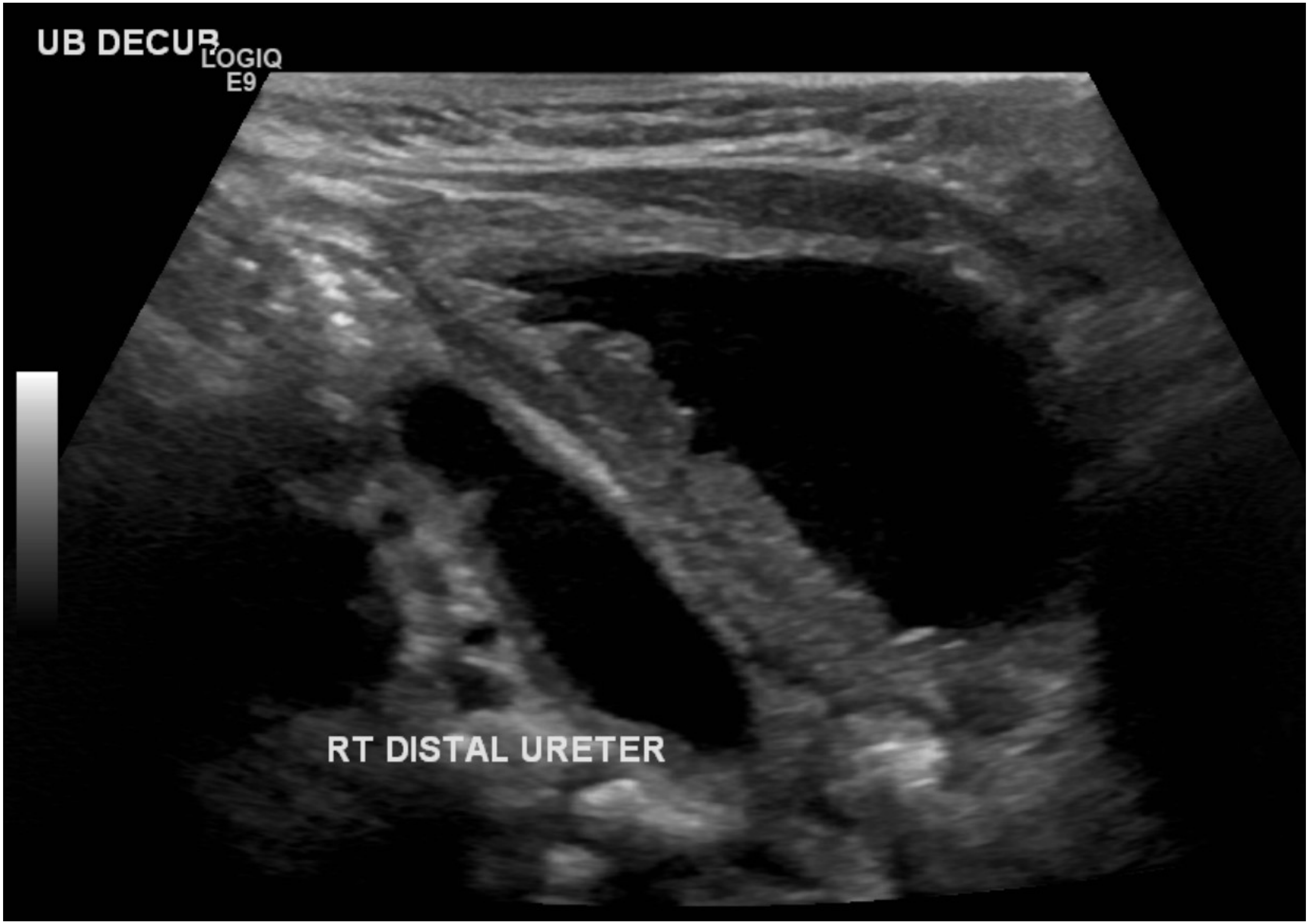
UB DECUB
LOGIQ
E9
RT DISTAL URETER

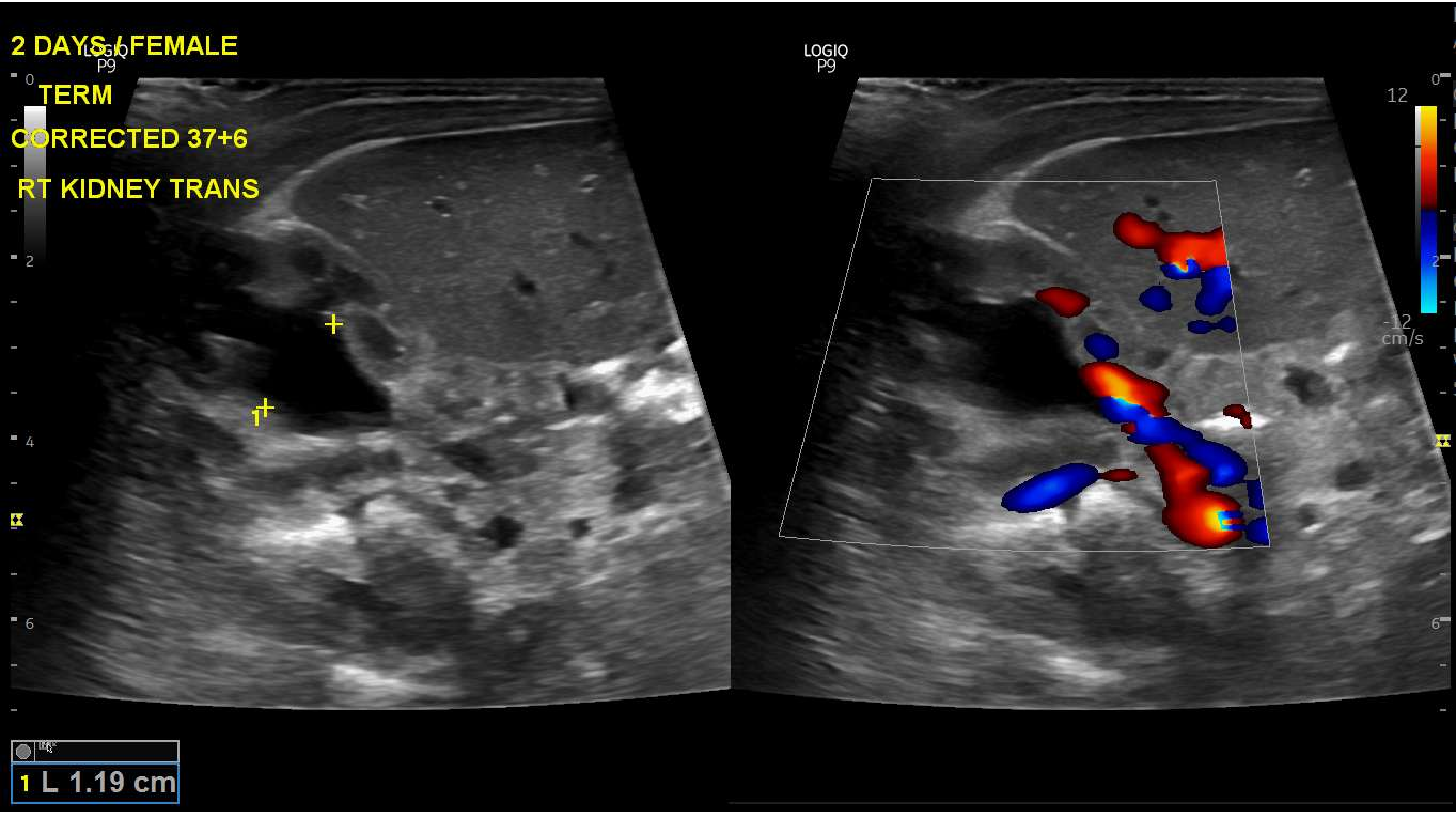

2 DAYS / FEMALE
TERM
CORRECTED 37+6
RT KIDNEY TRANS
LOGIQ
P9
LOGIQ
P9
1 L 1.19 cm
12
-12
cm/s

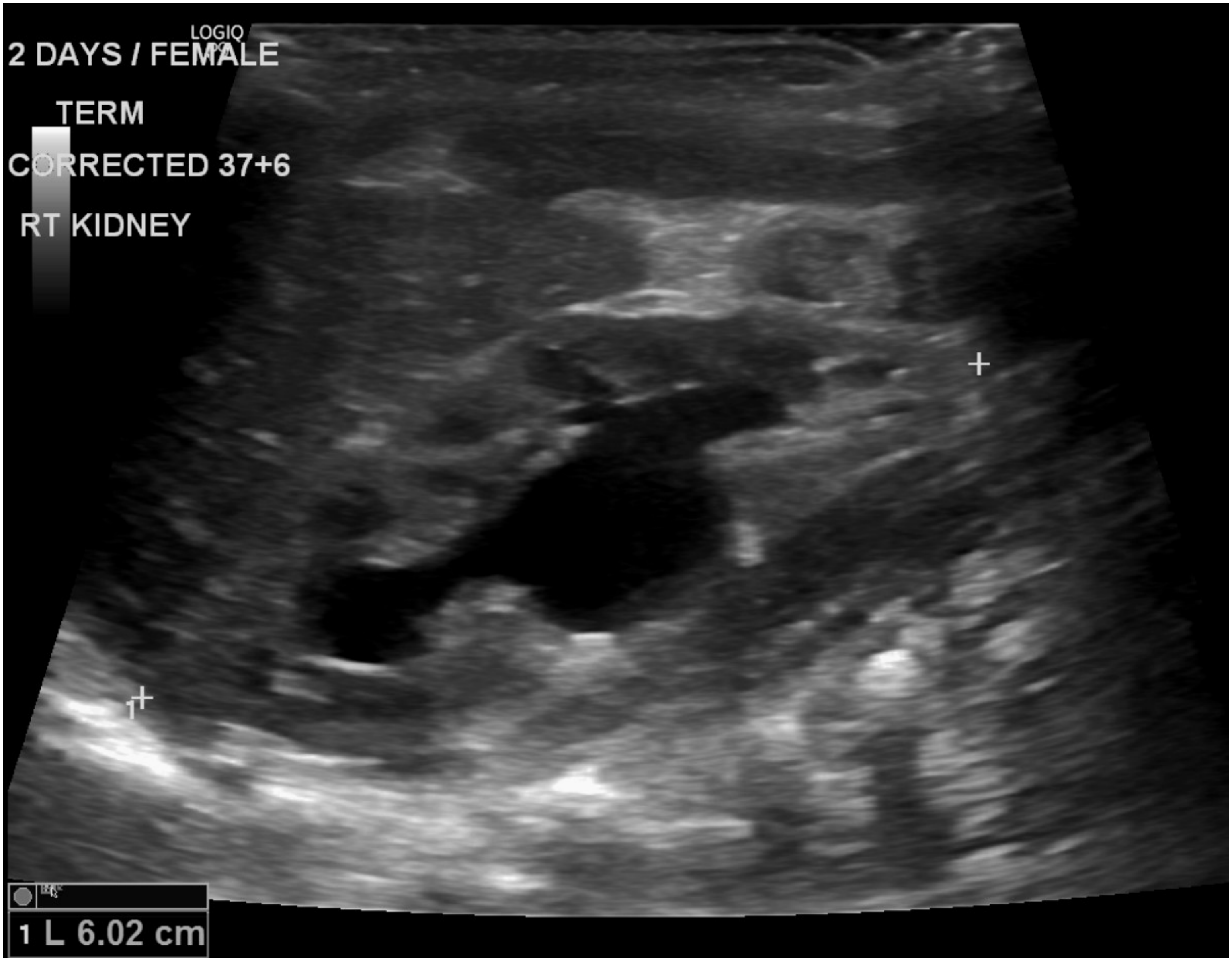
LOGIQ
2 DAYS / FEMALE
TERM
CORRECTED 37+6
RT KIDNEY
1 L 6.02 cm

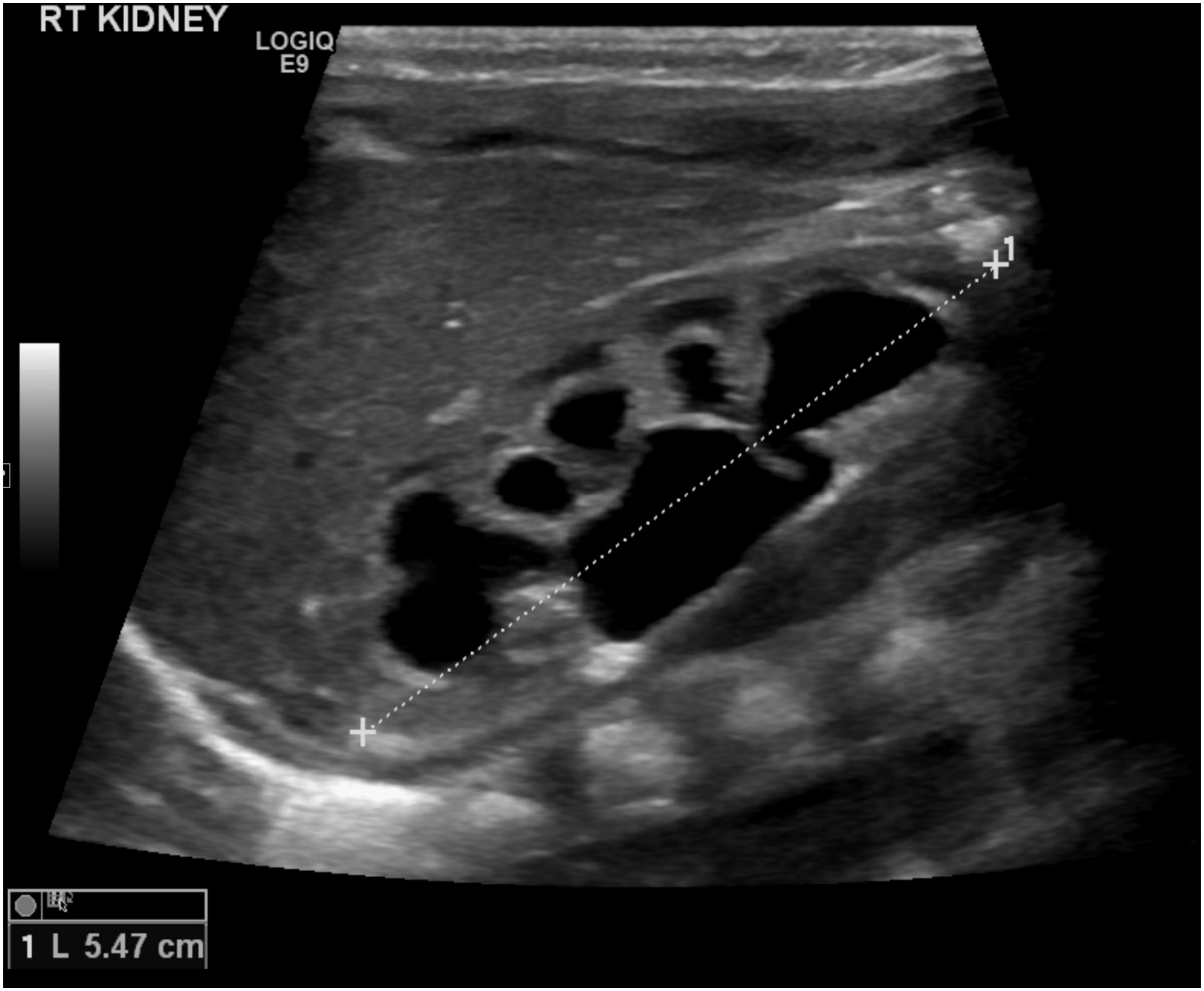
RT KIDNEY
LOGIQ
E9
1
1 L 5.47 cm

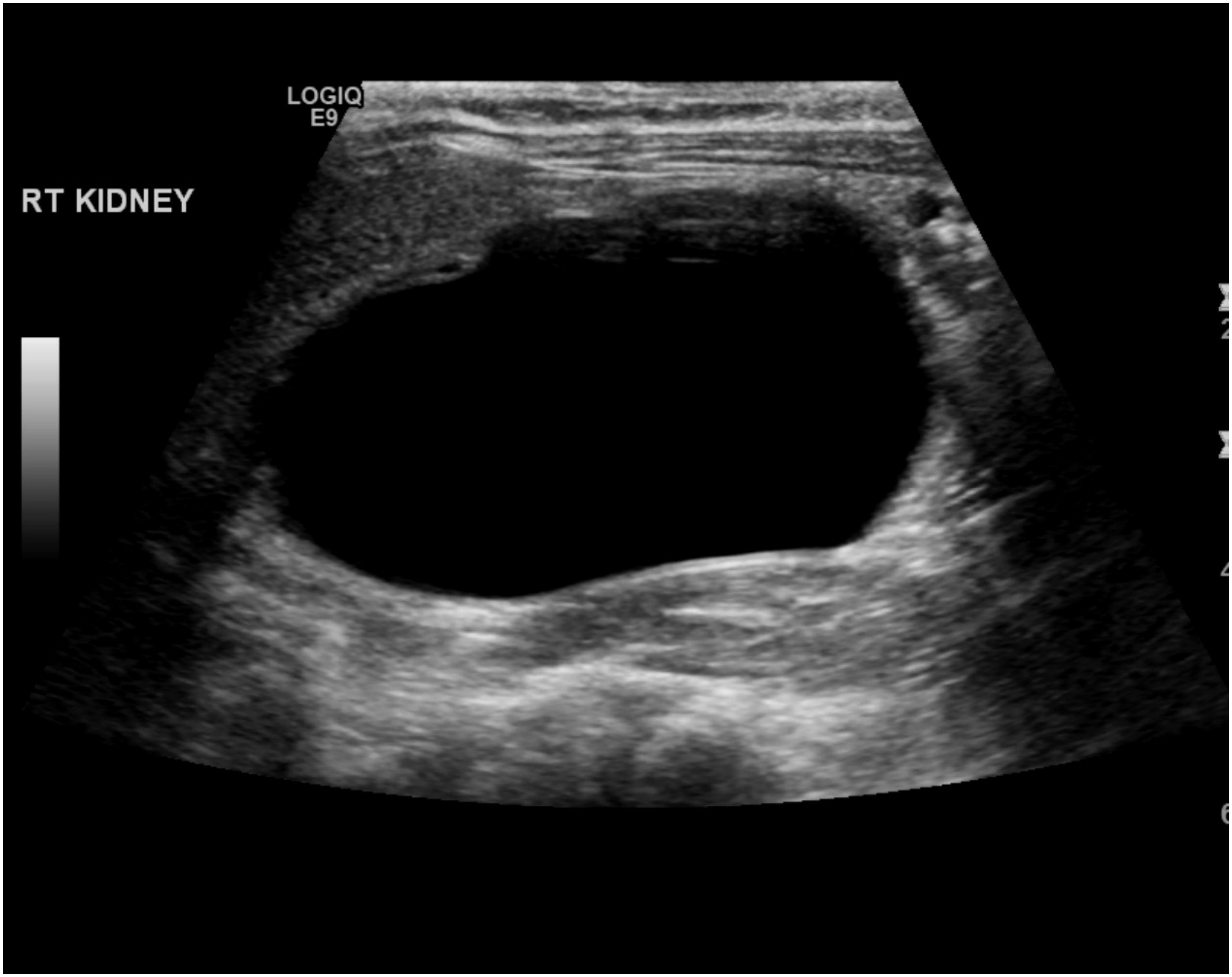
LOGIQ
E9
RT KIDNEY

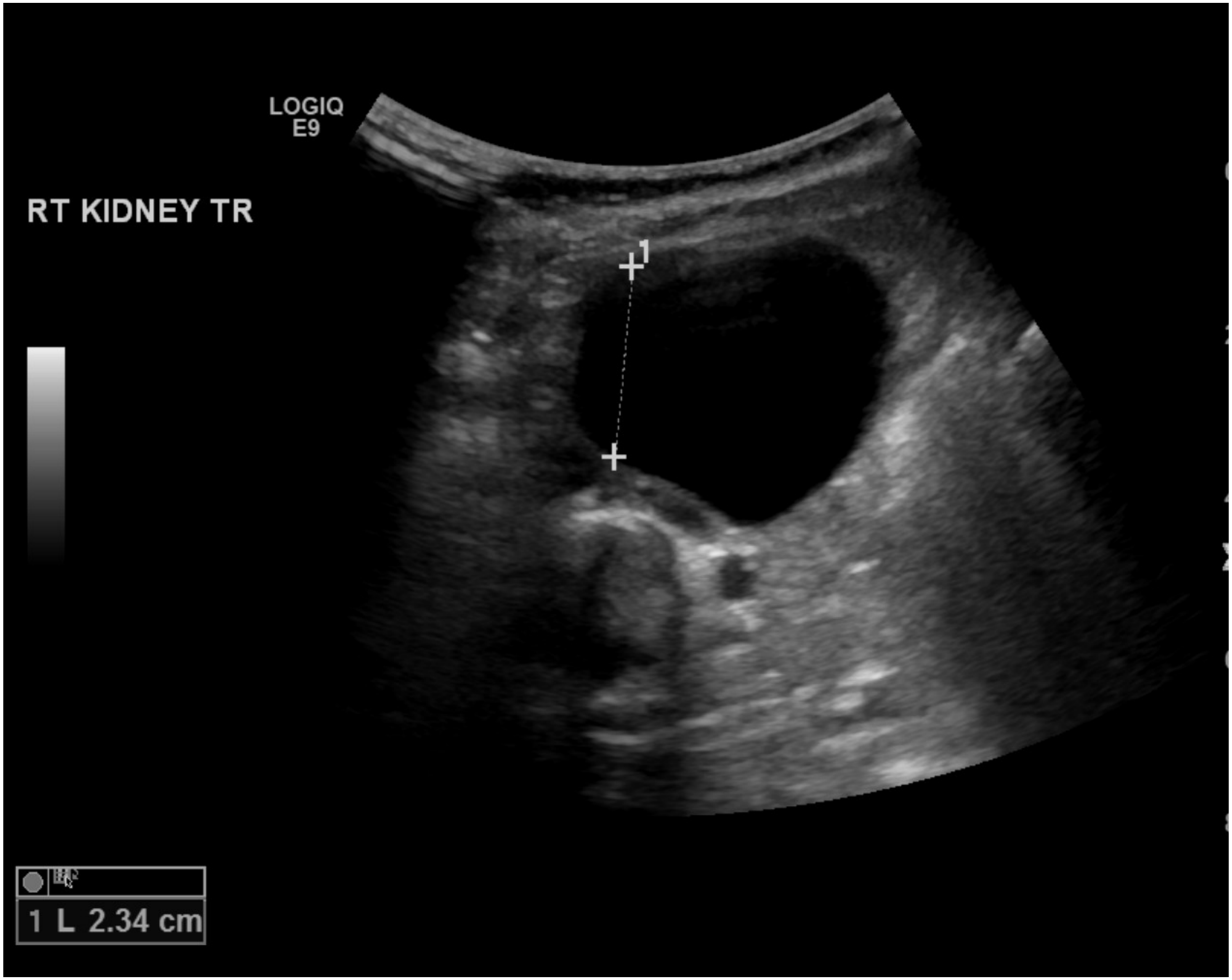
LOGIQ
E9
RT KIDNEY TR
1
1 L 2.34 cm

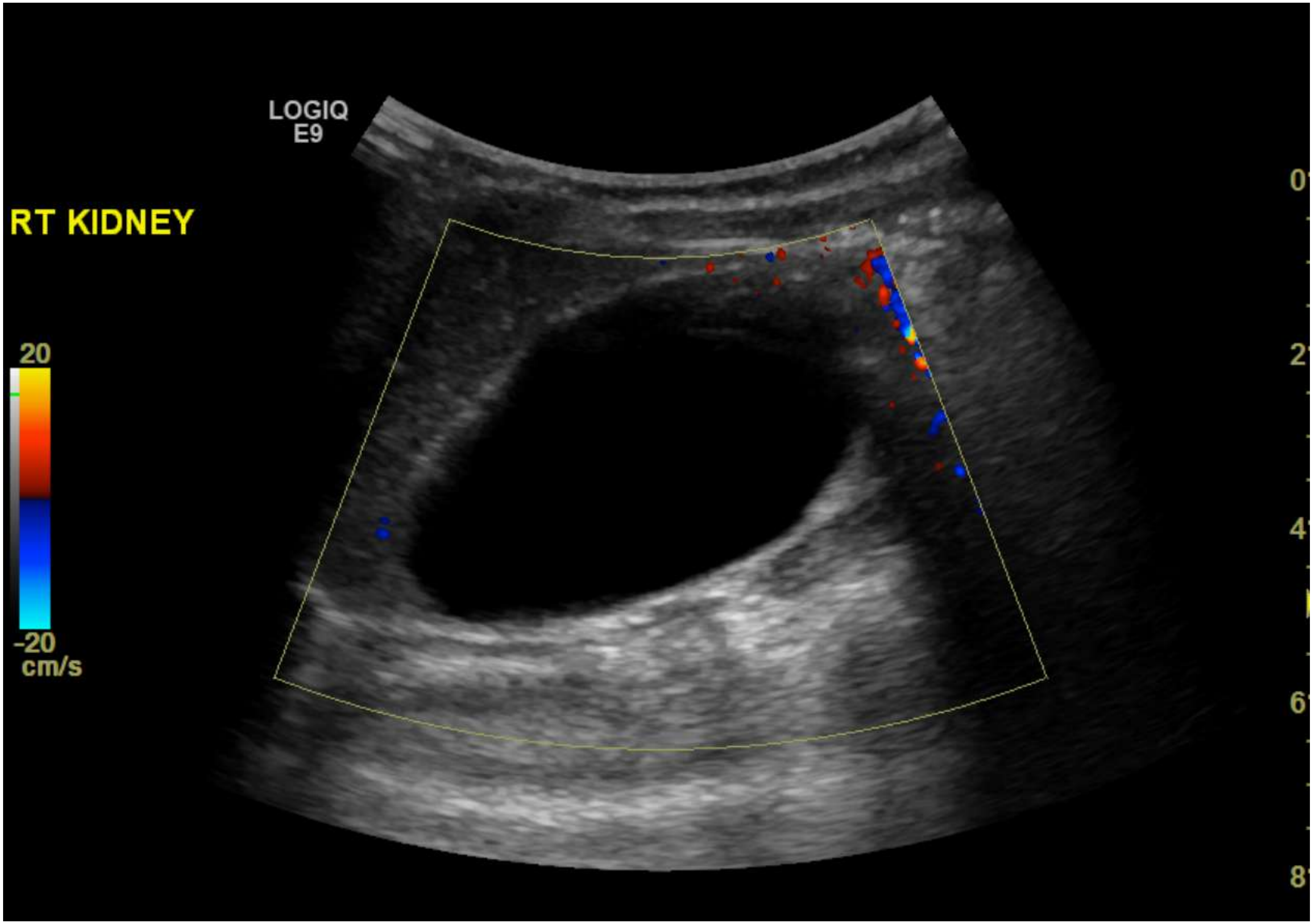
LOGIQ
E9
RT KIDNEY
20
-20
cm/s

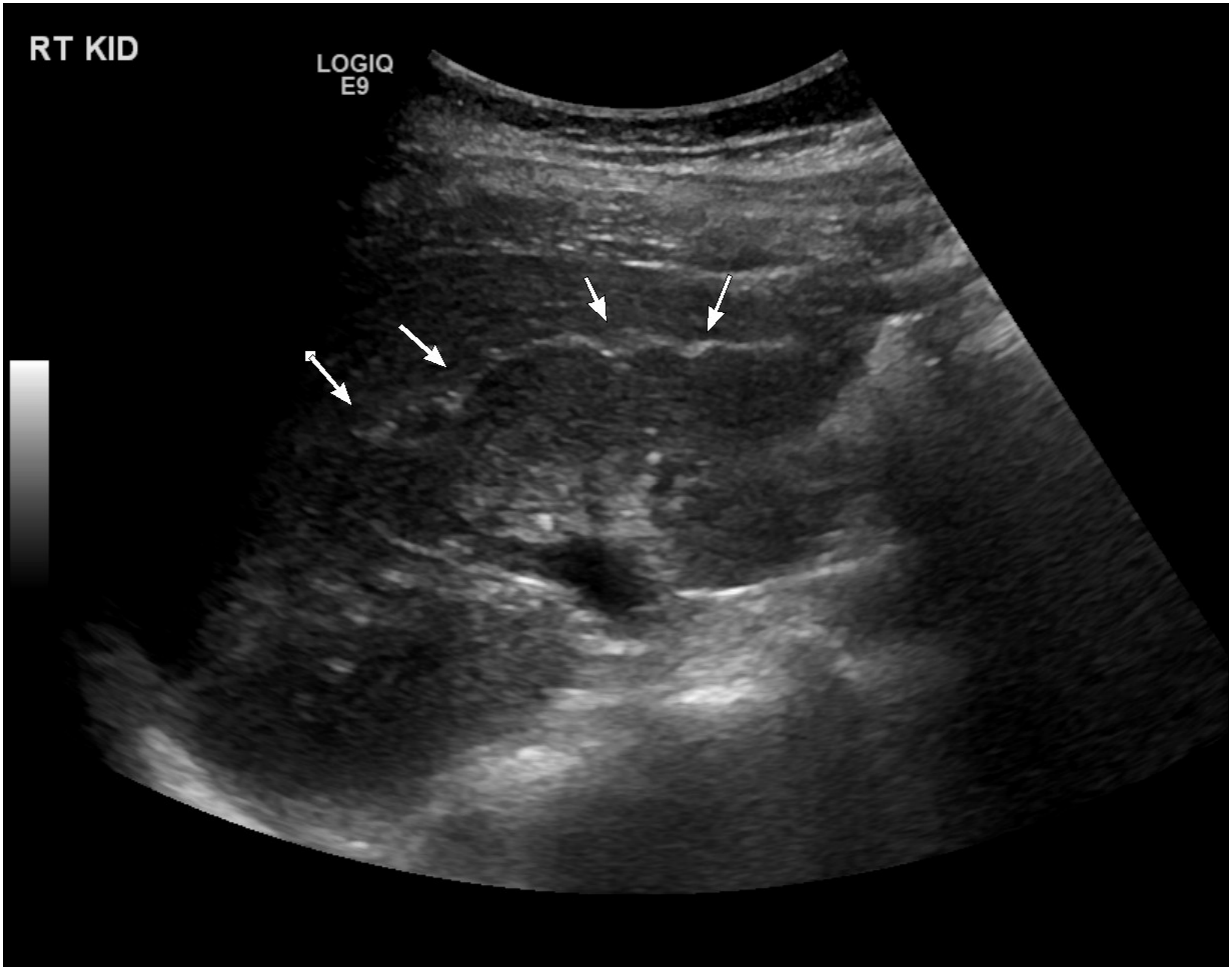
RT KID
LOGIQ
E9

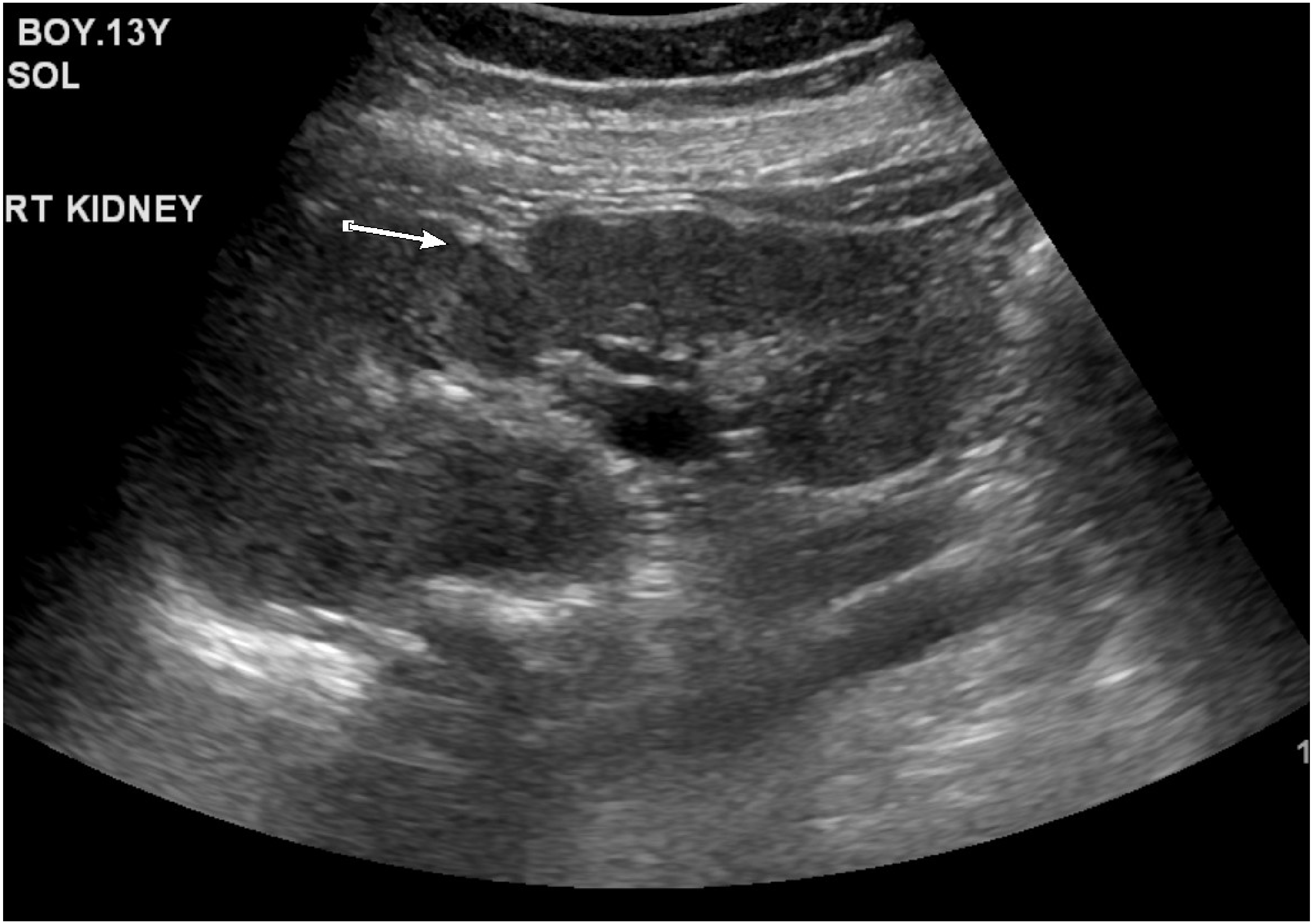
BOY.13Y
SOL
RT KIDNEY
1

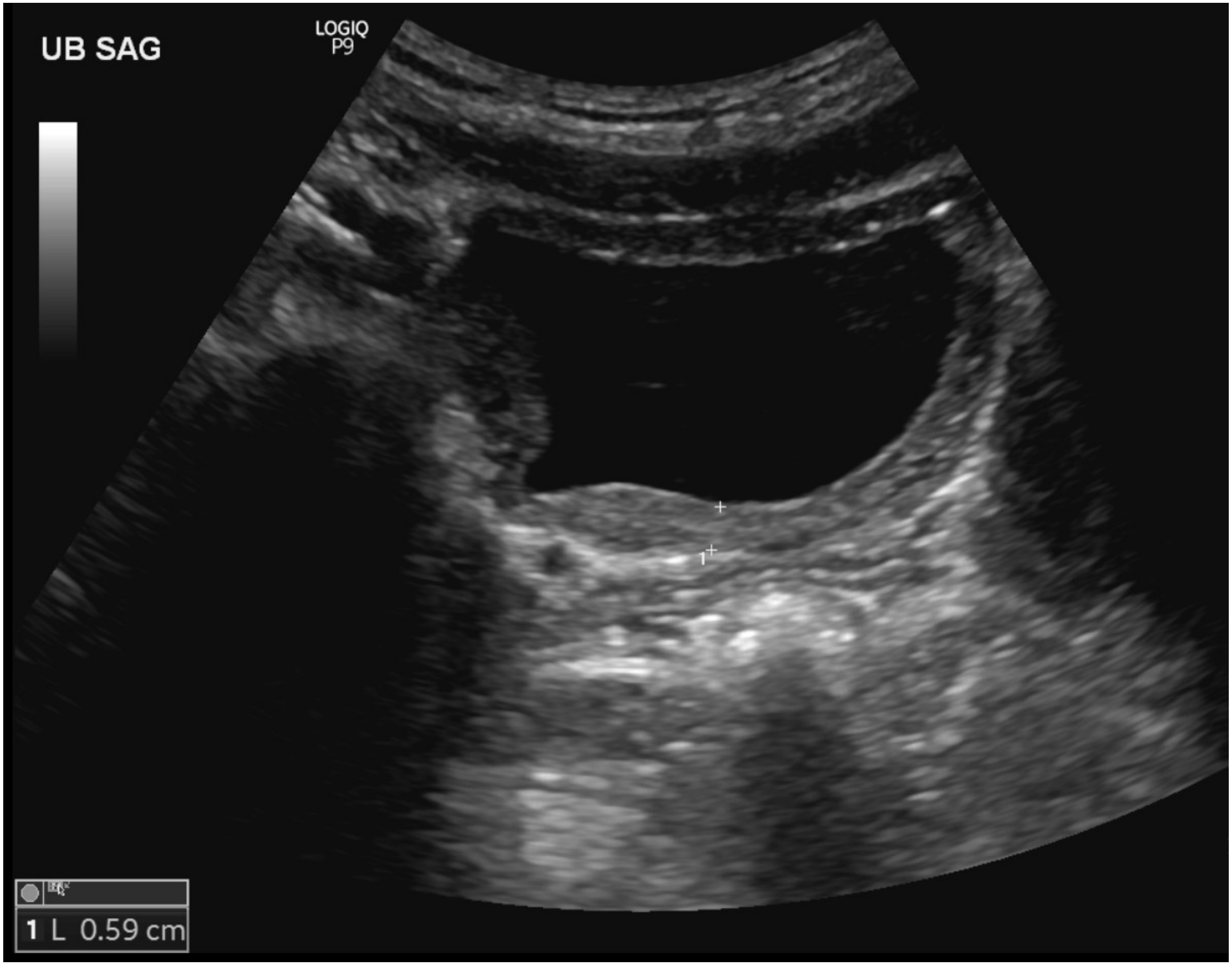
UB SAG
LOGIQ
P9
1 L 0.59 cm

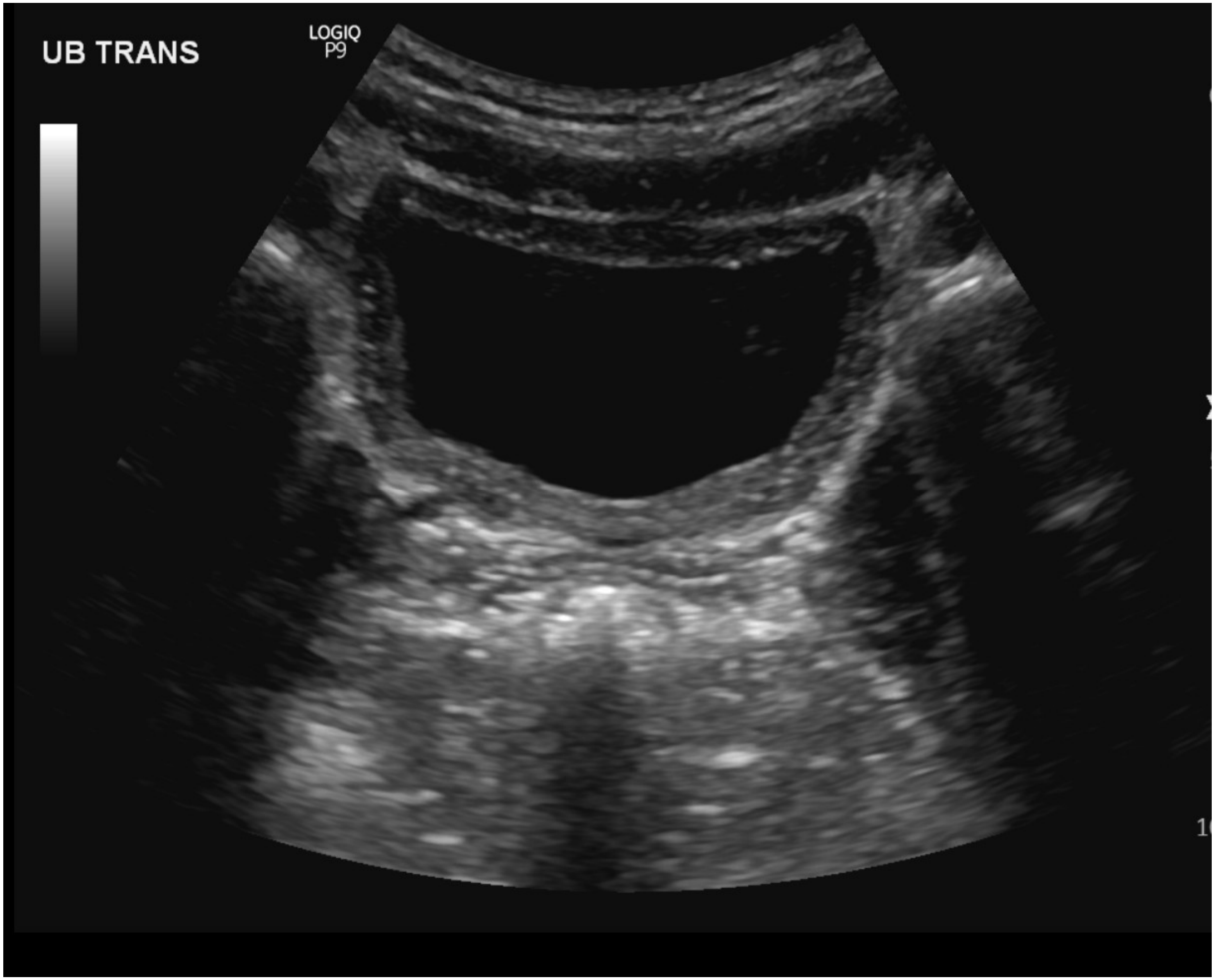
UB TRANS
LOGIQ
P9
